# Reducing misdiagnoses and cognitive errors using virtual patients and automated feedback in a clinical reasoning curriculum

**DOI:** 10.1186/s12909-026-08647-4

**Published:** 2026-01-28

**Authors:** Jason Waechter, Anita Kusnoor, Kara Eickman, David Smith, Matt Wong, Suzanne Rogers, Chel Hee Lee

**Affiliations:** 1 Department of Critical Care Medicine, University of Calgary, Calgary, Canada; 2 Department of Neurology, University of North Dakota School of Medicine and Health Sciences, Grand Forks, USA; 3 Department of Medicine, Baylor College of Medicine, Houston, USA; 4 Department of Medicine, Loyola Stritch School of Medicine, Chicago, USA; 5 Department of Academic Family Medicine, University of Saskatchewan, Saskatchewan, Canada; 6 Department of Primary Care, Idaho School of Osteopathic Medicine, Boise, US; 7 Department of Mathematics and Statistics, Department of Critical Care Medicine, University of Calgary, Calgary, Canada

**Keywords:** Misdiagnosis, Clinical reasoning, Curriculum, Deliberate practice, Pre-clerkship, Cognitive errors

## Abstract

**Introduction:**

Diagnostic errors remain prevalent across all specialties, driven largely by deficits in clinical reasoning (CR). Although CR is a core competency, most medical schools lack structured pre-clerkship CR training. Virtual patients (VPs) with automated feedback offer scalable, simulation-based training to improve diagnostic skills and reduce faculty workload. The aim of this study was to assess whether a CR curriculum using VPs with automated scoring and deliberate practice improves diagnostic accuracy and CR.

**Methods:**

We conducted a multi-site observational study across five North American medical schools. First- and second-year students completed up to 20 diagnostic VP cases on TeachingMedicine.com, each with automated scoring to inform individualized feedback. We analyzed 1.55 million datapoints from 12,400 cases completed by 1,066 students to assess differences in CR performance between correctly and incorrectly diagnosed cases, associations between CR components and diagnostic accuracy, and learning gains over time.

**Results:**

Misdiagnoses occurred in 20.1% of cases. Correct diagnoses were associated with higher diagnostic justification (DxJ) scores (+ 50%), better test ordering (+ 51%), and fewer cognitive errors (–89%). Multivariate analysis identified DxJ and cognitive errors as the strongest predictors of diagnostic accuracy. With repeated practice, students improved DxJ by 72%, test ordering by 40%, and reduced misdiagnoses threefold and cognitive errors by half, with no plateau observed after 20 cases. By end of pre-clerkship, first-year students who completed 20 cases outperformed second-year students who completed 10 in all CR metrics. All results were statistically significant with *p* < 0.0001.

**Conclusions:**

This curriculum shows that CR skills are highly trainable through deliberate practice. Improved DxJ and reduced cognitive errors are strongly associated with lower misdiagnosis rates. In contrast to a common misperception, training CR diagnostic skills is successful when started in the beginning of 1st year medical school prior to students’ acquisition of significant medical knowledge.

**Supplementary Information:**

The online version contains supplementary material available at 10.1186/s12909-026-08647-4.

## Introduction

Clinical reasoning (CR) can be defined as the cognitive processes and decisions that inform diagnoses and treatment plans; they include hypothesis generation and refinement, problem representation, data acquisition and interpretation, diagnostic justification and therapeutic decision making [1]. CR is a core competency of graduating medical students and is required for safe patient care [2].

However, despite increasing widespread awareness of the importance of CR, diagnostic errors caused by CR errors continue to be unacceptably high and occur across all specialties [3–5].

It is *expected* that students’ CR skills will improve significantly during clerkships. However, research suggests that clerkship improvements are less than those seen in pre-clerkship years [6]. CR deficits persist throughout training and even after graduation [7]. A recent international scoping review found that CR is rarely trained or explicitly assessed in health professions education programs [8]. A survey of U.S. medical schools highlighted the urgent need to strengthen clinical reasoning curricula, noting that most students began clerkships with limited understanding of key CR concepts and often received no formal preclinical training [9]. There is a well-recognized lack of CR training and assessment in health care education; only about 25% of schools have such a curriculum [9, 10].

Diagnostic CR is a trainable skill in pre-clerkship [11, 12]. However, beliefs that CR skills cannot be trained among students with low medical knowledge is a barrier to implementing training early in medical school. There are many calls to start CR training for pre-clerkship students and continue throughout all phases of undergraduate medical education [9, 13, 14]. Using common diseases with typical presentations, this training would increase formative feedback and self-reflection opportunities and enable diagnostic skill assessment and development prior to clinical rotations.

Faculty workload, faculty expertise, time requirements, and financial cost are all barriers to implementing CR skill development curricula [15, 16]. Frequent practice and feedback for large cohorts generates excessive assessor cognitive overload and burnout. Virtual patients with automated scoring that informs detailed individual feedback overcomes these barriers by minimizing faculty workload, thereby reducing the number of faculty required and faculty training.

A virtual patient (VP) is a computer based simulation and is suggested as an ideal platform to train CR [17–20]. VPs enable flexible cases, a safe learning environment, and can focus training on key CR concepts such as building a differential diagnosis (Ddx), revising the Ddx, collecting data, and identifying the correct diagnosis [21]. This may be especially true in the pre-clerkship environment, compared to the clerkship environment where these skills are taught and developed through live patient encounters.

VPs have been both recommended and empirically validated for training and assessing CR in pre-clerkship students [22]. Studies show that VP-based training can improve data gathering and enhance overall CR performance [21]. To achieve these benefits, however, VP activities must incorporate key instructional elements such as feedback, repetition, and sufficient case variety [19, 23]. When these conditions are met, and particularly when VP systems are combined with automated scoring and feedback, CR instruction can be scaled efficiently: a single faculty member can support repeated deliberate practice for more than 200 students, generating 100–150 data points per student per case in under five hours of faculty time per month.

These instructional design features align closely with principles of simulation-based CR training, which emphasizes structured learning supported by feedback and reflection to reduce reasoning errors and improve performance [24]. In this context, deliberate practice, characterized by iterative cycles of repeated performance and targeted formative feedback, has been demonstrated as a particularly effective framework for developing CR skills [25].

We are not aware of any educational intervention, with or without VPs, that reduce misdiagnoses in the pre-clinical years of medical school. The aim of this study was to evaluate whether a multi-site pre-clerkship CR curriculum using VPs and automated scoring to provide deliberate practice improves diagnostic accuracy and diagnostic clinical reasoning. We previously reported psychometric outcomes from a single-site, longitudinal diagnostic CR curriculum, which improved students’ Ddx, diagnostic justification (DxJ), and investigation scores, though not diagnostic accuracy [12]. For the current study, we expanded implementation to four additional medical schools, spanning both organ-based and traditional curricula, where students learn normal physiology in the first year and disease in the second.

Our study had three research questions. First, we sought to ascertain performance differences between components of diagnostic CR between correctly and incorrectly diagnosed cases. These components included building a Ddx, DxJ, ordering tests, cognitive errors, and time spent. Secondly, we analyzed if and to what extent the components of CR performance were associated with diagnostic accuracy within a multivariate analysis. Lastly, we examined whether CR components, cognitive errors and diagnostic accuracy improved with repeated deliberate practice.

## Methods

This prospective multi-site observational study was approved by the Conjoint Health Research Ethics Board at the University of Calgary (REB19-0065), the University of North Dakota (UND) (IRB00001300), University of Saskatchewan (USask) (#1378), Loyola University Chicago Stritch School of Medicine (exempted), Baylor College of Medicine (H-53759), and Idaho College of Osteopathic Medicine (ICOM) (exempted). Clinical trial number: not applicable.

### Participants

Seven cohorts of first year (M1) and 3 cohorts of second year (M2) medical students from five medical schools completed multiple virtual patient CR cases with automated scoring that generated detailed individual feedback on TeachingMedicine.com/Dx as part of their mandatory curriculum. Students provided informed consent for data inclusion in research and received no compensation.

### School details

One of the schools was an osteopathic (DO) program, while the others were allopathic (MD) programs; all were 4 year programs. One school followed a “traditional” curriculum (normal physiology and anatomy in the first year, abnormal in the second), while the remaining four used “organ system–based” curricula, each with a different sequence of organ systems (see Appendix). Within the organ system-based curricula, case presentation order strived for 25% overlap with the current organ system and 75% with previously taught systems, so that learners could not assume that a given case correlated with their current area of study.

### Case creation

A seven-member committee representing internal medicine, critical care, neurology, cardiology, obstetrics, general surgery, and pediatrics identified at least three common diagnoses from 15 systems/categories, totaling 58 diagnoses. 23 cases with typical presentations resulted from this list. The cases and scoring rubrics are further detailed in the Appendix.

### Case completion by student

Students created an online account on TeachingMedicine.com using their name, email, and password. At each site, an introductory didactic explained the process of clinical reasoning, defined relevant terms, and demonstrated how to complete a sample case. Subsequently, first-year students received one case at a time, while second-year students received one or two; cases were equally spaced throughout pre-clerkship, every 3–4 weeks. Cases were completed during independent student time. We encouraged group work, internet searches, and use of textbooks. Case order differed between schools and their cohorts.

Each clinical scenario has four stages: a one-sentence introduction, history, physical exam, and investigations. At each stage, students create/revise a Ddx of up to five conditions and identify a most probable diagnosis when they navigate to the next stage. Students assign history or physical exam findings to each diagnosis to indicate whether the data increased or decreased its likelihood, thereby actively identifying pertinent positives and negatives. In the investigations stage, students are only provided tests results for tests they order and then use the test results to perform further DxJ as describe above. Students could order unlimited tests; each time they clicked “Get Results” it counted as one “round”, and they could complete as many rounds as they wished. Students navigated back and forth between stages as needed; case completion occurred when students identified the most probable diagnosis and their degree of certainty after the investigations stage. Further published details on this CR software are available [12, 26].

Students received individualized automated formative feedback for each completed case based on over 125 data points generated per case (see Scoring below). This iterative cycle of practice and feedback constituted deliberate practice. Feedback included quantitative and qualitative insights on correct and incorrect choices regarding: building a Ddx, performing DxJ, ordering and interpreting investigations, cognitive errors, and identifying the most probable diagnosis.

The VP platform used free-text entry for diagnoses and test ordering to increase diagnostic uncertainty and avoid the cueing effects of multiple-choice formats [27]. Predefined lists limit independent generation of ideas and encourage recognition over reasoning. Lists also eliminate the need to consider diagnoses or tests *not listed*, which undermines clinical reasoning. Equally important, test results were shown only if the learner ordered the test. Providing unsolicited positive results usually reveals the diagnosis and invalidates the reasoning process. Test selection is a critical skill that must be assessed directly because failure to order appropriate tests contributes to misdiagnosis [28].

Following completion of a case, students attended a one-hour whole-class review for each case. A faculty member conducted a PowerPoint-guided "think-aloud" demonstration while navigating the case, followed by a didactive PowerPoint lecture on CR and a review of class performance statistics and student questions.

### Scoring, cognitive errors, and penalty calculations

An algorithmic scorecard created by 2–3 experienced medical experts for each clinical scenario was used to automatically calculate scores. Diagnoses were classified as “appropriate” for the case, earning students a point if included in their Ddx. History, physical exam, and investigation data were categorized as “required,” “neutral,” or “wrong” for increasing or decreasing a diagnosis’s probability. DxJ points were: awarded for correctly assigning required data, not awarded if not assigned, or partially deducted for incorrect assignments. Investigations were labeled “required” or “inappropriate,” with points gained or lost accordingly. Students received full points for selecting the correct diagnosis and partial points if it was listed as their second choice. Percent scores were calculated for each of building a Ddx, DxJ, investigations, and final diagnosis.

Using DxJ, most probable Dx and Ddx data, we created individualized algorithms for identification of cognitive errors, including: failure to confirm correct diagnosis (premature closure), failure to rule out, confirmation bias, anchoring, and search satisficing (see Appendix for details). We assessed every case using these algorithms and identified when behaviors consistent with these cognitive errors were present and provided this feedback with an error count to the students.

Penalty calculations were performed by identifying a threshold (typically twice the mean) for maximum allowable number of tests and rounds of tests ordered; if exceeded, students lost points. They also lost points for not using results of tests ordered or for ordering inappropriate tests. These penalties were designed to inhibit learners from “gaming the system” by ordering tests indiscriminately.

### Statistical analysis

We compared correctly versus incorrectly diagnosed cases in both univariate and multivariate analyses for all components of CR described above for which students were provided scores, as well as case navigation details (time spent per stage, and number of times each stage was viewed). We determined mean CR scores, cognitive error counts, and diagnostic accuracy for up to 20 cases completed by students to determine the learning effect of the curriculum.

Data were analyzed using R version 4.0.1 (R Development Core Team, 2024). For comparisons between the correct and incorrectly diagnosed cases, we employed Wilcoxon tests for non-normally distributed data and t-tests for normally distributed data.

To assess the relationship between CR components and misdiagnoses, we used generalized estimating equations (GEE), a special form of logistic regression model, to account for the fact that students were clustered within schools and each student completed multiple cases. This method adjusts for differences between schools and that multiple cases for one student will be dependent on that student, but independent from other students. Because students completed cases in a fixed sequence, we used an autoregressive correlation structure, which assumes results from adjacent cases are more similar than those farther apart. Time variables were log-transformed, and highly skewed counts were converted to yes/no categories (Table 1). For learning-curve analyses, we used a generalized mixed-effects model and plotted the fixed-effect trends across case sequence.

**Table 1:**
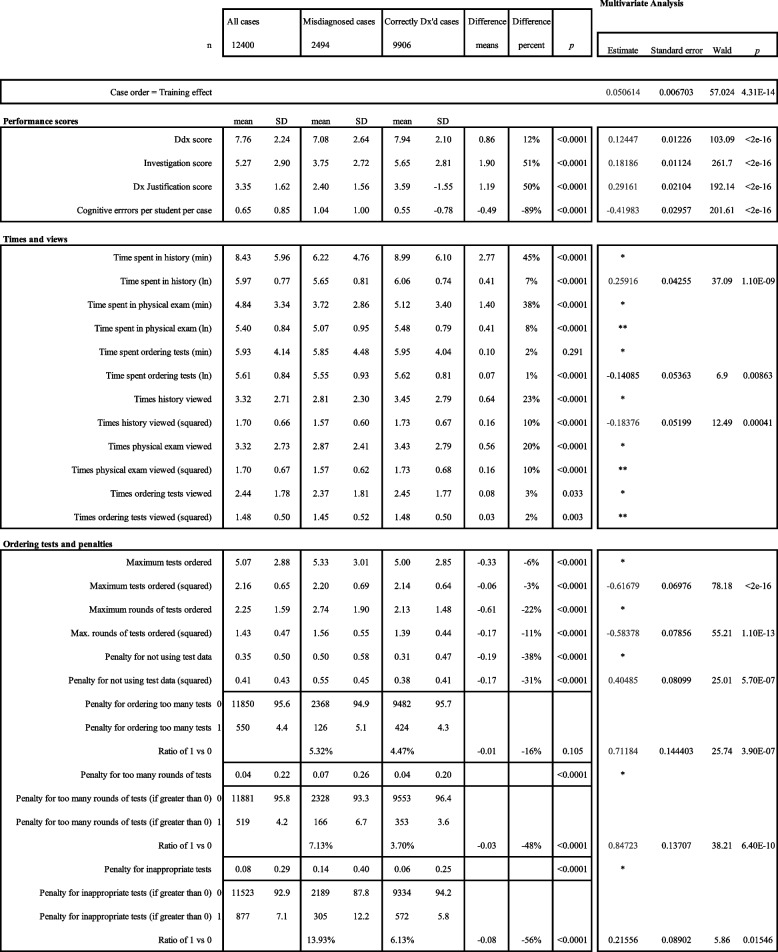
Scores for performance, time, navigation, and penalties among correctly and incorrectly diagnosed cases. Data is presented as bivariate analysis; multivariate analyses are on the right

*Not modelled, transformed **Not significant, removed

## Results

Between August 2021 and November 2024, 1,066 of 1,269 (84%) first- and second-year medical students consented to the research project and completed a total of 12,400 cases; students completed between 4 and 20 (mean = 13.6) cases. Three schools completed 18 or more cases. Students misdiagnosed 2,494 (20.1%) cases. Average data generated per completed case was 125, resulting in a dataset of 1.55 million data analyzed.

Our first research question queried differences among CR components of correctly vs. misdiagnosed cases (Table 1). A group comparison revealed that CR scores [mean(SD)] were higher among correctly diagnosed cases for: performing DxJ [2.4(1.6) vs. 3.5(1.6) = + 50%], ordering tests [3.8(2.7) vs. 5.7(2.1) = + 51%], and building a Ddx [7.1(2.6) vs. 8.0(2.1) = + 12%]. Among misdiagnosed cases, cognitive errors were 89% higher [1.0(1.0) vs. 0.56(0.78)] compared to correctly diagnosed cases. All above comparisons were significant with *p* < 0.0001.

Among correctly diagnosed cases, students spent more total time [minutes (SD)] reviewing the history [6.2(4.7) vs. 9.0(6.1) = + 45%] and physical exam [3.7(2.9) vs. 5.1(3.4) = + 38%], but not investigations, compared to misdiagnosed cases. Through navigating back and forth, they also visited the history [2.8(2.3) vs 3.5(2.8) = + 23%] and physical exam [2.9(2.4) vs. 3.4(2.8) = + 20%] stages more often, with only a negligible increase in investigation views among correctly diagnosed cases. Correctly diagnosed cases involved fewer [5.3(3.0) vs. 5.0(2.9) = –6%] tests and fewer [2.7(1.9) vs. 2.1(1.5) = –22%] test rounds. Penalties were also lower among correctly diagnosed cases for: not using test data [0.50(0.58) vs. (0.31(0.47) = –38%), excessive tests [5.3% vs. 4.5% = –16%], excessive test rounds [7.1% vs. 3.7% = –48%), and inappropriate tests [14% vs. 6.1% = –56%). All above comparisons were significant with *p* < 0.0001.

**Fig. 1:**
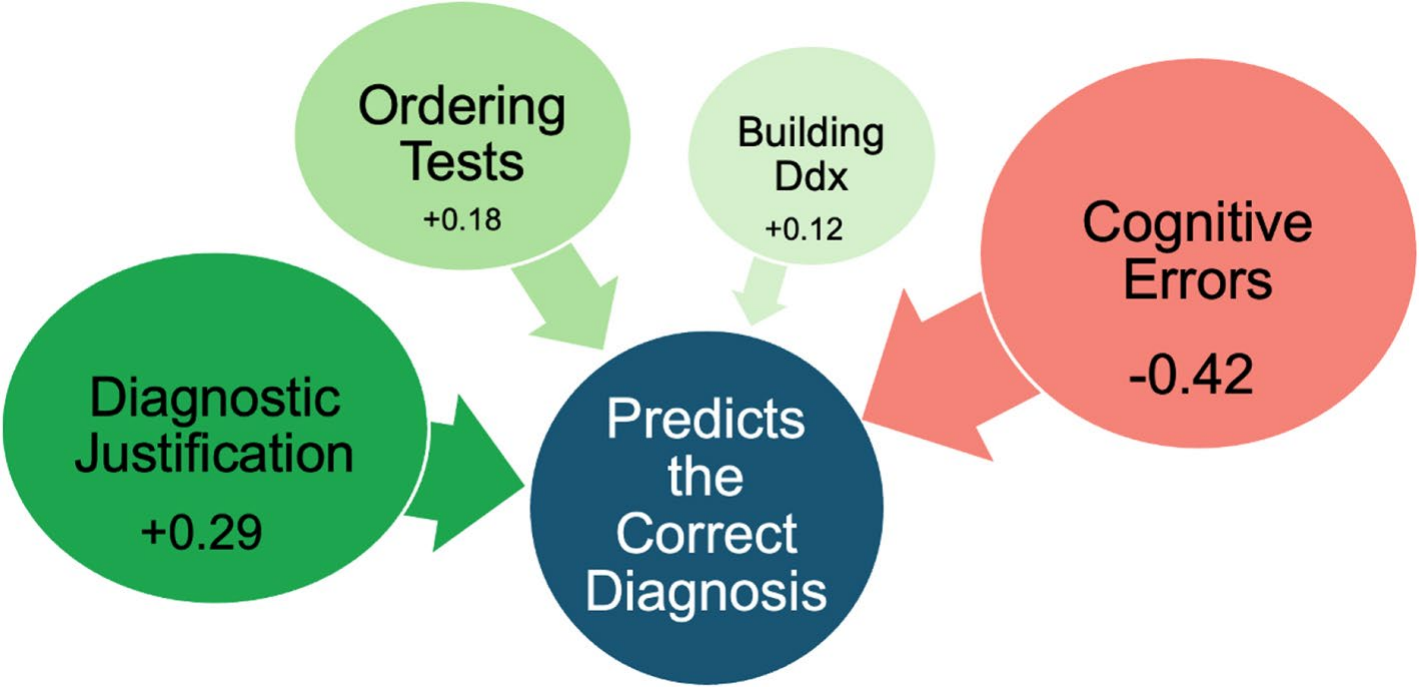
Components of Diagnostic Reasoning that Predict the Correct Diagnosis. The GEE analysis identified predictors of correct diagnosis. The strongest predictor was fewer cognitive errors, followed by higher performances in: diagnostic justification, appropriate test ordering, and building a Ddx. Since many cognitive errors were defined using algorithms based on specific diagnostic justification patterns, diagnostic justification was much more strongly predictive than ordering tests or building the Ddx

To assess our second research question if CR components were associated with misdiagnoses, we performed a multivariate logistic analysis (Table 1). Positive estimates predicted correct diagnoses; negative values predicted misdiagnoses [estimate(SE)]. DxJ [+ 0.29(0.02)] and cognitive errors [–0.42(0.03)] showed the strongest associations with diagnostic accuracy; test ordering [+ 0.18(0.01)] and building a Ddx [+ 0.12(0.01)] also had significant, though smaller, effects (Fig. 1, *p* ≪ 0.0001).

**Fig. 2:**
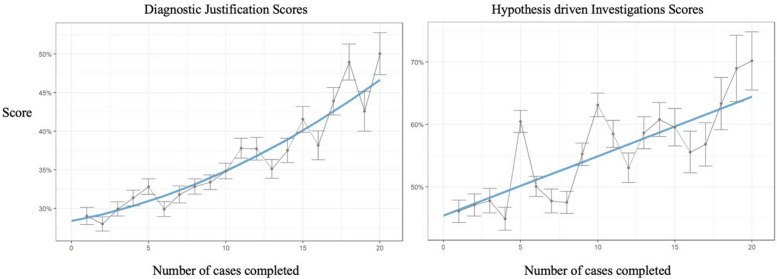
The learning curves for diagnostic justification and hypothesis driven ordering of tests. After completing 20 cases with formative feedback, scores for diagnostic justification increased from 27% to 46.5% (a 72% increase) and scores for ordering tests increased from 46% to 64.5% (40% increase). The slopes of both graphs do not flatten out at the end of pre-clerkship, suggesting that more training in clerkship would result in higher performance

Our third research question examined whether scores for building a Ddx, DxJ, ordering investigations, cognitive errors, and diagnostic accuracy improved with deliberate practice. Over 20 cases, scores for building a Ddx increased slightly from 79% to 90.7% (10.7%), while DxJ improved from 27% to 46.5% (+ 72%), and test ordering from 46% to 64.5% (+ 40%) (Fig. 2, *p* < 0.0001).

**Fig. 3:**
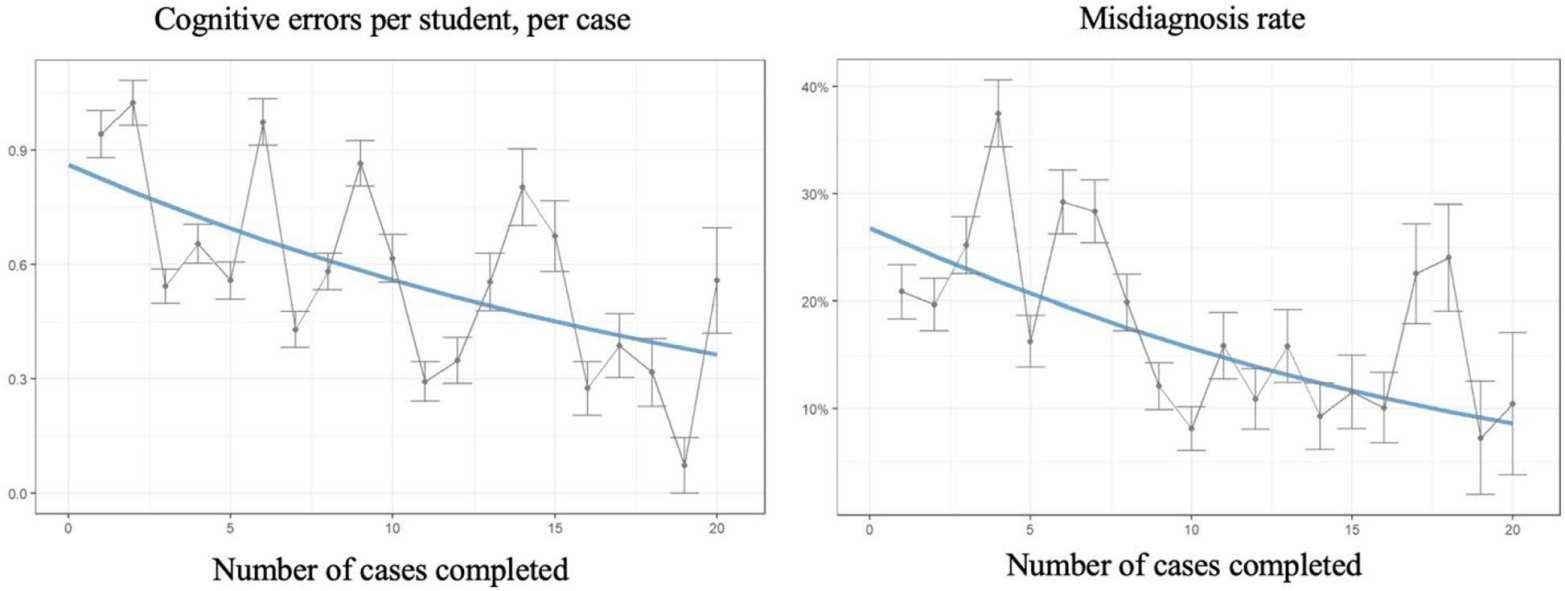
Cognitive errors and misdiagnoses are reduced with targeted deliberate feedback provided for every case. Cognitive errors occurred at a rate of 0.87 per student per case at the start of the curriculum, indicating that nearly every student experienced a cognitive error for every case. This reduced by 50% to 0.43 errors per student per case. Rates of misdiagnoses were 27% at the start of the curriculum and 9% at the end of the curriculum, indicating a threefold reduction of misdiagnoses

Cognitive errors dropped by half from 0.87 to 0.43 per student per case, and misdiagnoses fell threefold from 27% to 9.0% (Fig. 3, *p* < 0.0001). Learning curves did not plateau for any of the psychometric variables at 20 cases.

**Fig. 4:**
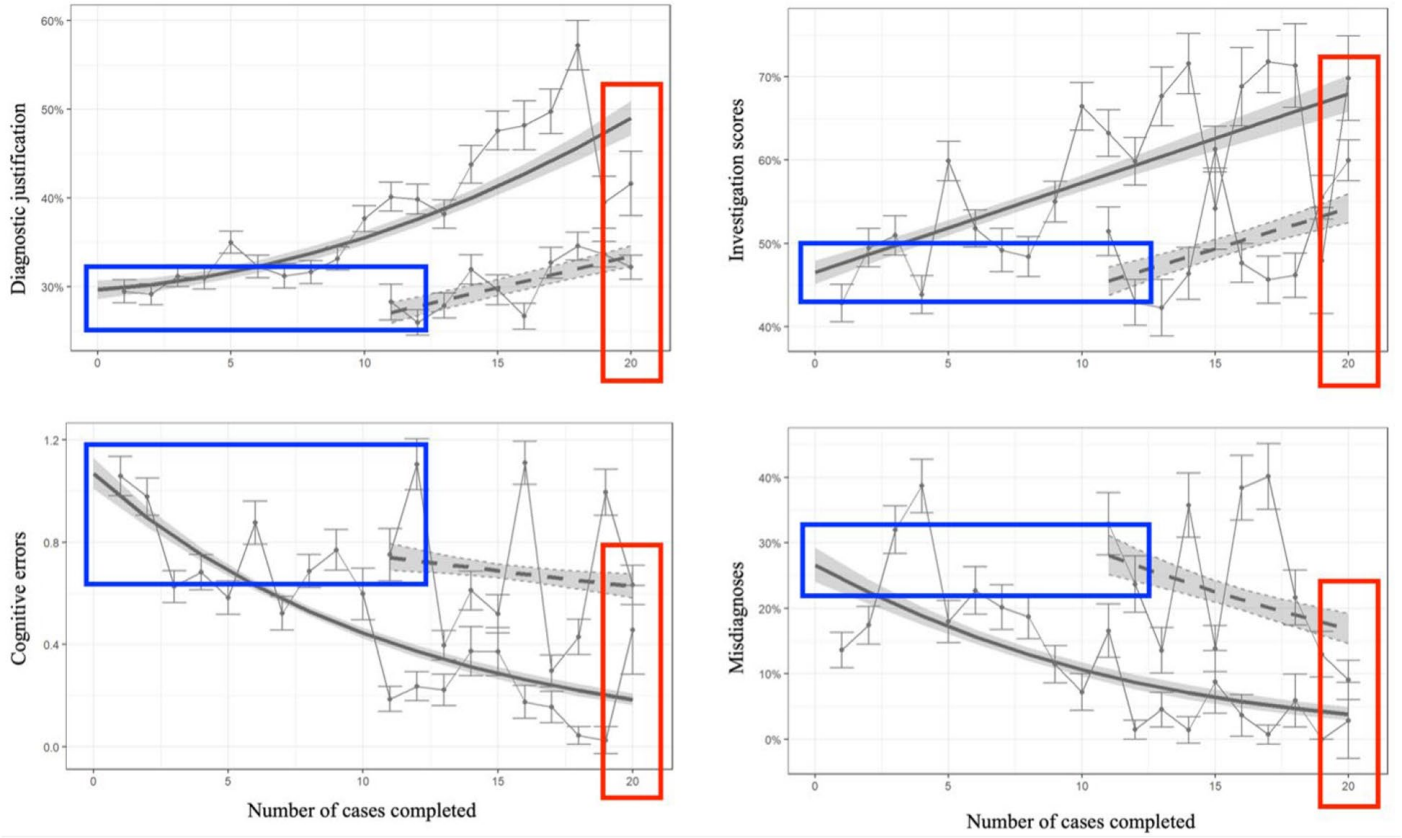
Comparison of M1 versus M2 students at start of curriculum and end of pre-clerkship. At the start of the curriculum (blue rectangles), diagnostic reasoning and misdiagnosis rates were similar between M1 (solid lines) and M2 (dashed lines) students but M2 students demonstrated fewer cognitive errors. By the end of pre-clerkship (red rectangles), M1 students who completed 20 cases outperformed M2 students who completed 10 cases in all 4 metrics

We compared the seven M1 cohorts with the three M2 cohorts who did not use the curriculum in their first year of study. At the *start* of the curriculum, scores for performing DxJ, ordering investigations, and misdiagnosis rates were not significantly different between the M1 and M2 groups, but M2 students did demonstrated fewer cognitive errors. At the *end of pre-clerkship*, the M1 and M2 cohorts completed 20 and 10 cases respectively. The M1 cohort outperformed the M2 cohort at the end of pre-clerkship for all of: performing DxJ, ordering investigations, misdiagnosis rates, and rates of cognitive errors (Fig. 4, *p* < 0.0001).

## Discussion

The aim of this study was to assess whether a pre-clerkship CR curriculum using VPs with automated scoring to provide deliberate practice improves diagnostic accuracy and clinical reasoning. Our first two research questions explored CR performance differences between correctly and incorrectly diagnosed cases. Univariate analyses showed that correct diagnoses were associated with fewer cognitive errors and higher scores in DxJ, test ordering, and Ddx building. Further, students who diagnosed correctly spent more time analyzing the history and physical exam, ordered fewer tests, and incurred fewer test ordering-related penalties.

Multivariate logistic analysis identified higher DxJ scores and fewer cognitive errors as the strongest independent predictors of diagnostic accuracy, with smaller but significant contributions for scores for test ordering and Ddx building. Three of the five cognitive error algorithms were based upon special cases of DxJ (see Appendix); thus, the 2 most predictive factors for diagnostic accuracy were overall DxJ scores, and special cases of DxJ.

DxJ is increasingly recognized as a challenging aspect of clinical reasoning, with deficits observed even in students who make correct diagnoses and these deficits persist into clerkship [11, 12, 29–31]. DxJ is a key component of diagnostic clinical reasoning that strongly predicts graduate exam performance, yields high item discrimination, and improves assessment reliability [29, 31]. Our results combined with previous publications underscore the urgent need for CR curricula to prioritize deliberate practice in DxJ, a critical yet often underemphasized skill [29, 30].

Our third research analysis showed that scores for building a Ddx, performing DxJ and ordering investigations, as well as avoiding cognitive errors and misdiagnoses all improved with deliberate practice across both years of pre-clerkship (Figs. 2 and 3). Our findings align with prior studies showing DxJ is trainable with dose–response improvements through simulation and feedback [11, 12].

Improvements in diagnostic reasoning were attributable to the CR curriculum. At baseline, M2 students with no prior exposure to the curriculum performed similarly to M1 students in diagnostic justification, test ordering, and diagnostic accuracy. This provides evidence that CR skill development did not occur in the absence of the curriculum. By the end of pre-clerkship, the M1 cohort who completed 20 cases significantly outperformed the M2 cohort who completed only 10 in DxJ, ordering tests, avoiding cognitive errors, and diagnostic accuracy, underscoring the value of increased practice before clerkship.

None of the performance or learning curves plateaued at 20 cases. This suggests that substantial skill development would be expected to continue into clerkship, reinforcing previously published calls for longitudinal four-year curricula [10, 13, 25, 32].

There are many barriers to implementation of a CR skills training curriculum, namely faculty workload, faculty expertise, limits to curricular time, and a belief that it cannot be trained early [10, 15, 32, 33]. Workload and faculty expertise issues are resolved using VPs and computer algorithms that generate data, automate scoring and provide feedback to the students.

Our results are relevant to educators and researchers because: 1) misdiagnoses are strongly linked to weaknesses in diagnostic justification and cognitive errors, underscoring the need for CR curricula to focus more on these diagnostic reasoning processes; 2) deliberate practice improves diagnostic reasoning and reduces misdiagnoses in 1st year medical students, contrary to common assumptions; 3) these improvements do not occur in the absence of deliberate practice; 4) VPs with automated scoring and feedback overcome faculty workload, training, and expertise barriers and provide a feasible method to scale deliberate practice to large cohorts and; 5) our learning curves did not plateau, lending evidence to calls for 4 year longitudinal CR training programs.

Strengths of our study include its large multi-site dataset, comprising 1.55 million data points; this might be the largest CR psychometric outcomes study to date. The high student consent rate (84%) increased the likelihood that the study population was highly representative of the total population. The high number of completed cases (up to 20) increased the generalizability and reduced the relative error variance of scores introduced by case variation [10, 13, 25, 32]. We implemented the CR curriculum into both traditional and organ-based curriculum schools, providing evidence of seamless integration into a variety of curricula. The curriculum included CR components that aligned with published CR competencies, including Ddx building, DxJ, test ordering, and identifying a leading diagnosis [13, 34, 35].

The scope of this work was limited in that we did not assess information gathering through history-taking or physical examination. AI-driven history-taking tools were unavailable when we began, and virtual patients only poorly simulate physical exams. Still, separating data collection from analysis may be advantageous, as these distinct skills can be assessed independently, and junior students may struggle to perform both simultaneously.

Other limitations of our study include the lack of comparison with performance data from other assessment methods as well as the simulated nature of the curriculum. It remains unclear how well the findings translate to real-life clinical environments.

In conclusion, our multi-site, classroom-based pre-clerkship clinical reasoning curriculum utilized virtual patients and automated scoring to address common barriers to CR implementation. We showed that clinical reasoning skills are highly trainable through deliberate practice and that improved diagnostic justification and reduced cognitive errors are strongly associated with lower misdiagnosis rates. Finally, in contrast to a common misperception, we demonstrated that implementation of a CR curriculum is successful for CR skill development at the start of 1 st year medical school prior to students’ acquisition of significant medical knowledge.

## Supplementary Information

Supplementary Material 1.

## Data Availability

All data available upon request.
