# Supplementary material for: Reducing misdiagnoses and cognitive errors using virtual patients and automated feedback in a clinical reasoning curriculum
Source: BMC Med Educ. 2026 Jan 28;26:414. doi: 10.1186/s12909-026-08647-4 (PMC12983517; doi:10.1186/s12909-026-08647-4)
Supplement: Supplementary file 1 — Supplementary Material 1. [file 12909_2026_8647_MOESM1_ESM.docx]

**Reducing Misdiagnoses with Virtual Patients in Clinical Reasoning Education**

Appendix (Supplemental information)

**List of Cases: (sorted by age of patient).** These are the demographics, chief complaint, and diagnosis, with misdiagnosis rate for all simulated cases used in the curriculum None of these cases represent real patients.

| **Age** | **Gender** | **Chief complaint** | **Diagnosis** | **Misdiagnosis rate** |
| --- | --- | --- | --- | --- |
| 10 | M | Rash | ITP | 25.6% |
| 20 | M | Chest pain | Pericarditis | 3.1% |
| 23 | F | Abdo pain | C diff. colitis | 71.3% |
| 23 | F | Abdo pain | Pelvic inflammatory disease | 43.4% |
| 24 | F | Chest pain | Pulmonary embolism | 20.6% |
| 25 | Non-binary | Knee pain | ACL injury | 1.7% |
| 30 | M | Nausea | Hepatitis B | 13.7% |
| 30 | M | Unconscious | Bacterial meningitis | 13.1% |
| 31 | F | Abdo pain | Ectopic pregnancy | 18.9% |
| 34 | F | Weight loss | Hyperthyroidism | 16.9% |
| 34 | F | Weight loss | Depression | 0.0% |
| 35 | F | Cough | Bacterial pneumonia | 21.8% |
| 35 | F | Hand tingling | Carpal tunnel syndrome | 4.9% |
| 35 | F | Headache | Migraine | 29.2% |
| 40 | F | Cough | Sarcoidosis | 21.3% |
| 40 | F | Headache | Secondary HTN | 4.4% |
| 42 | F | Weakness | Multiple sclerosis | 18.4% |
| 47 | M | SOB | Colon cancer | 62.2% |
| 54 | F | Syncope | Aortic stenosis | 35.7% |
| 55 | M | Abdo pain | Gastric ulcer | 12.5% |
| 55 | M | Back pain | Ankylosing Spondylitis | 19.9% |
| 60 | M | Chest pain | Pericarditis | 15.0% |
| 70 | M | Abdo pain | AAA | 6.1% |

**School Details:** the participating schools, their cohorts by year of graduation, and curriculum details

| School/Grad year | Curriculum | Course Type | Students consented | Cases completed |
| --- | --- | --- | --- | --- |
| USask 2027 | Organ system | Clinical Reasoning | 106 | 4 |
| UND 2024 | Organ system | Clinical Skills,  “Clinical Reasoning Workshops” | 57 | 11 |
| UND 2025 |  |  | 62 | 20 |
| UND 2026 |  |  | 43 | 20 |
| UND 2027 |  |  | 67 | 13 |
| Baylor 2027 | Organ system | Clinical Skills | 215 | 9 |
| Loyola 2025 | Traditional | Clinical Skills | 80 | 13 |
| Loyola 2026 |  |  | 164 | 18 |
| ICOM 2026 | Organ System | Clinical Skills | 104 | 18 |
| ICOM 2027 |  |  | 156 | 10 |

University of North Dakota (UND), University of Saskatchewan (USask), Loyola University Chicago Stritch School of Medicine, Baylor College of Medicine, and Idaho College of Osteopathic Medicine (ICOM).

**Example of Case:** [Click here](https://www.teachingmedicine.com/dx/) to do a case. To watch video for Case Walkthrough, click the “How to do a Case” button and for video that explains stats and feedback, click the “Scoring and stats” button.

**Introduction stage:** Students must enter at least 1 diagnosis to proceed to the history. Diagnoses are entered by free text and match to a database of > 6,000 diseases. There is a limit of 5 Dx that can be added to the Ddx. Dx’s in the Ddx can be deleted during all stages of the case if required.


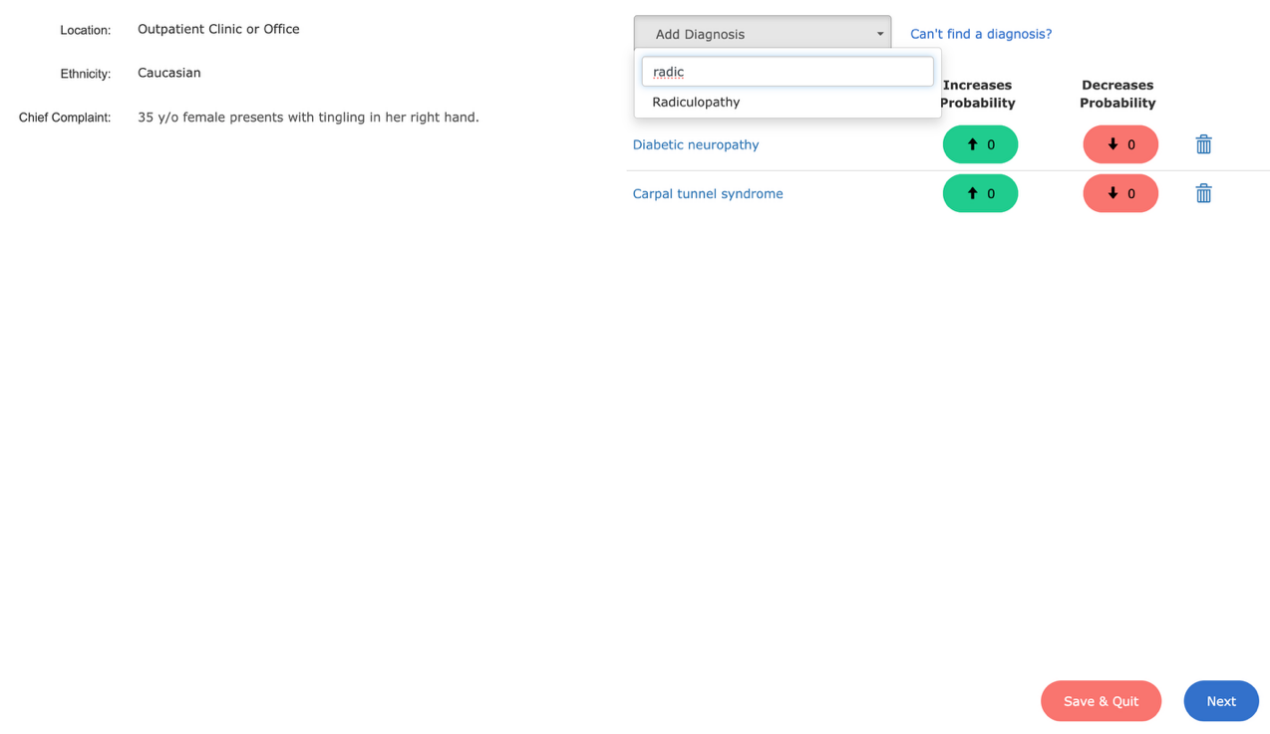


On completion of each stage, students are asked what their most probable diagnosis. If they navigate back and forth between stages, they are NOT asked this question again.

**
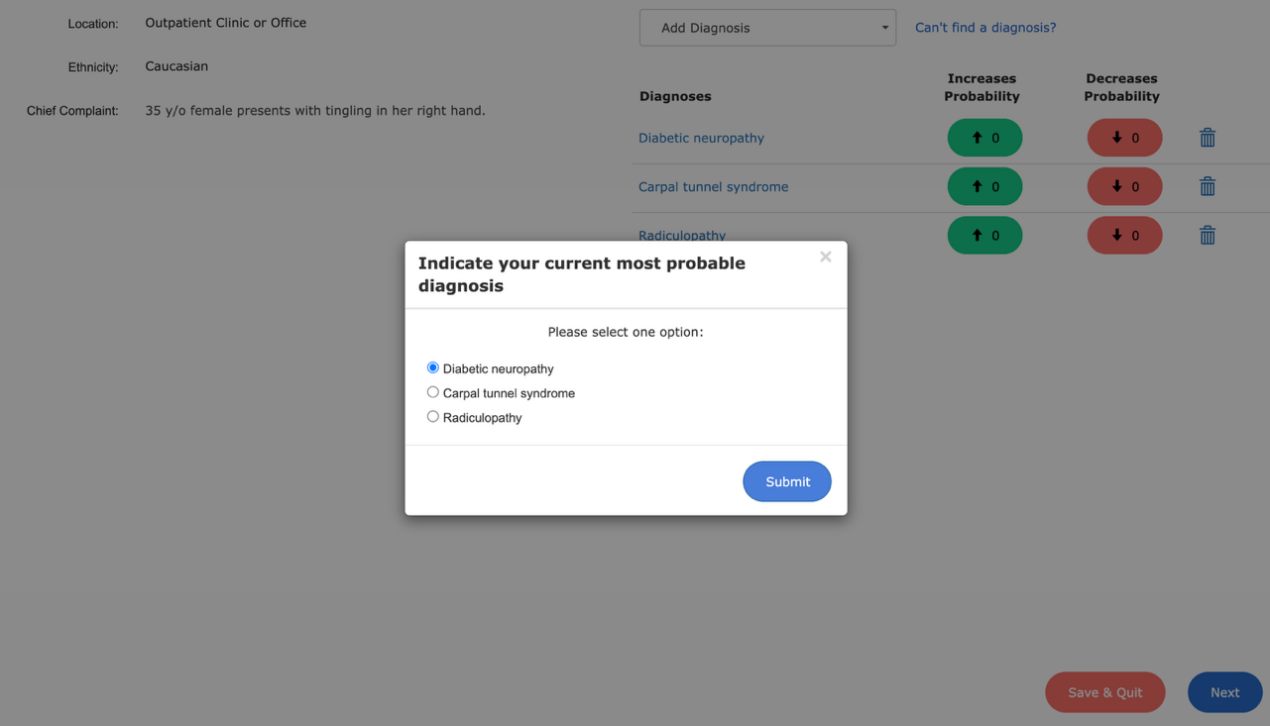
**

**History stage:** Highlighted data in the history is clicked and assigned as increases probability (green button) or decreases probability (red button) for each diagnosis (when appropriate).


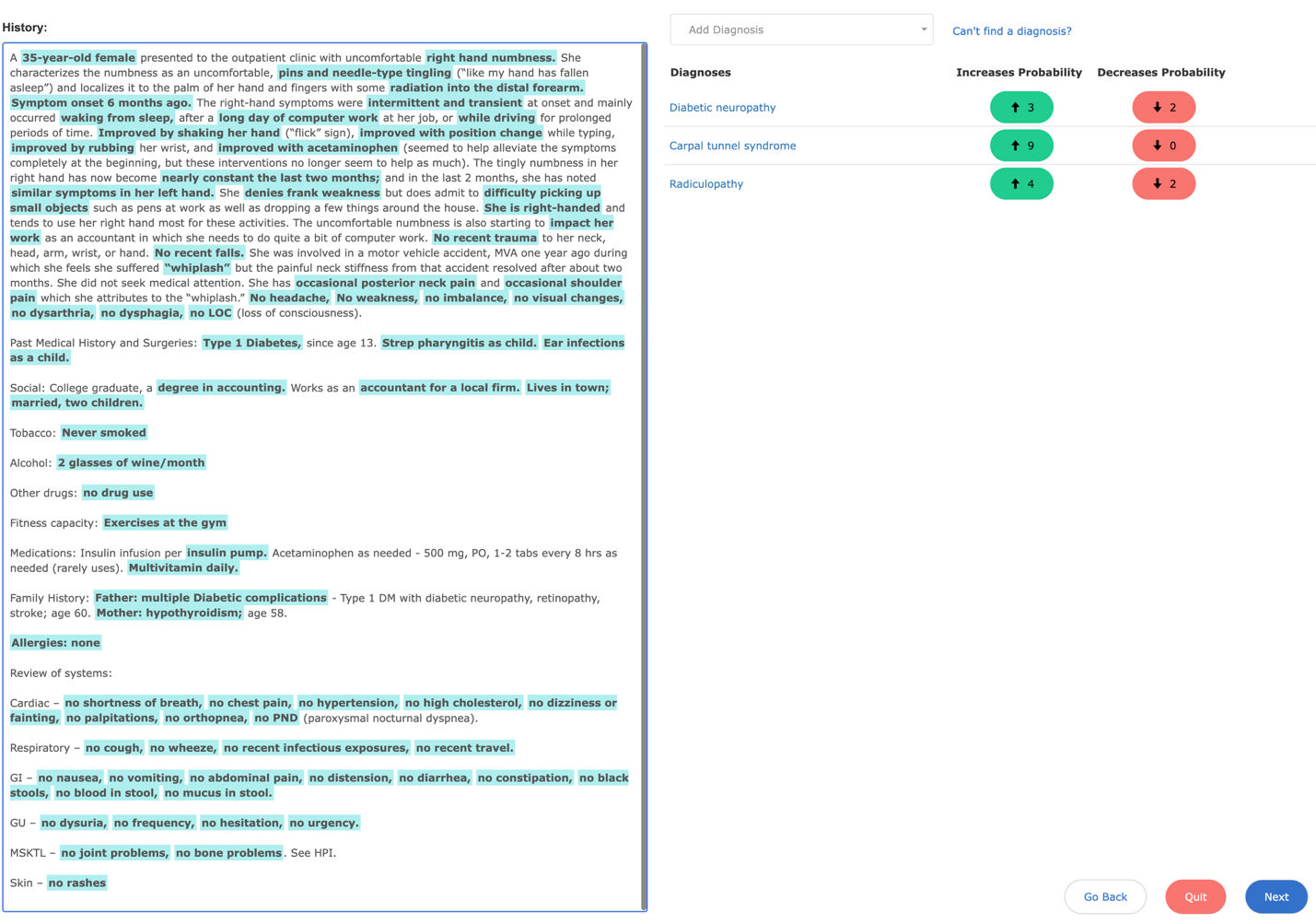


**Physical Exam Stage:** The physical exam stage is identical to the history stage, but with the details of the physical exam instead of the history.


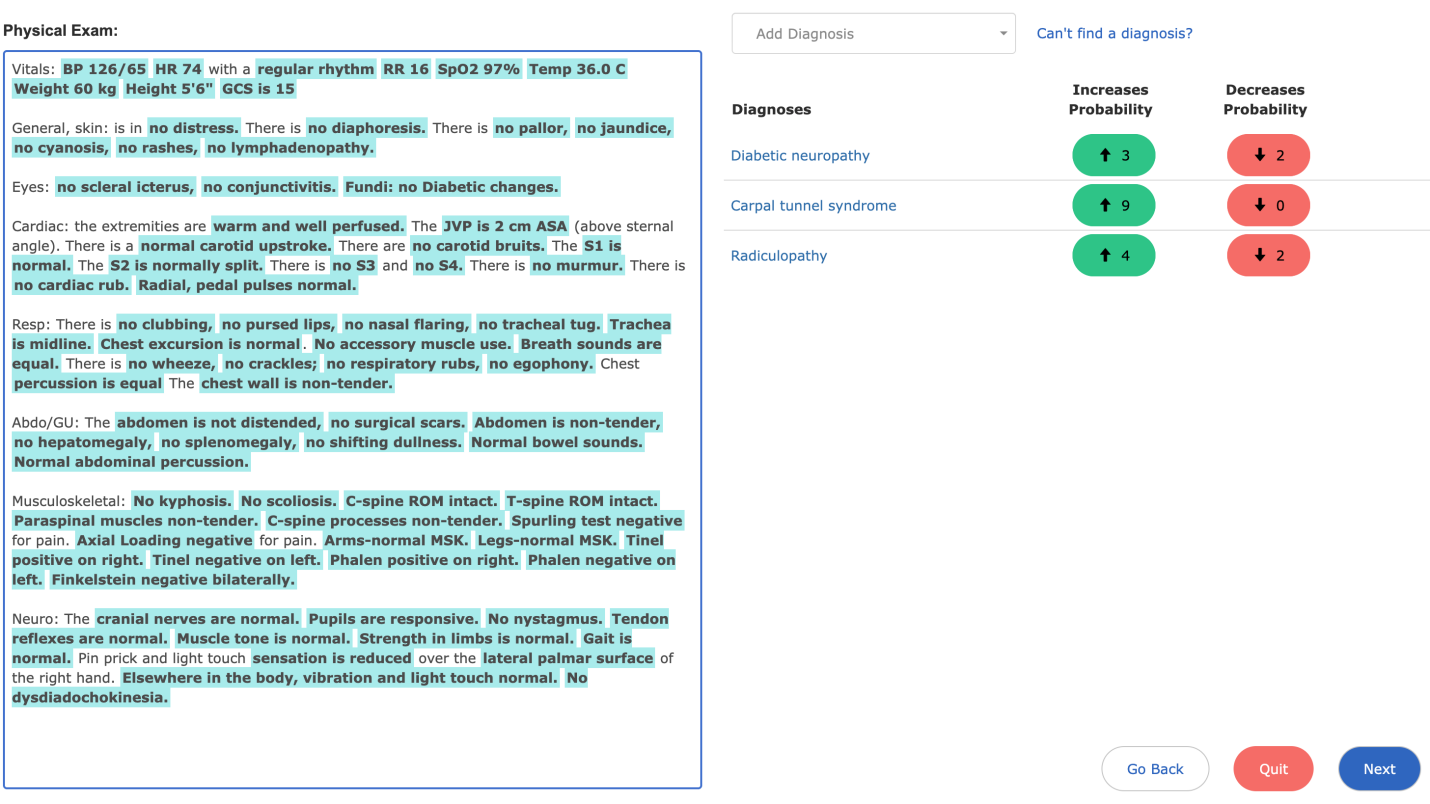


**Investigations Stage:** this stage starts with no test results (screenshot is from a headache case):

**
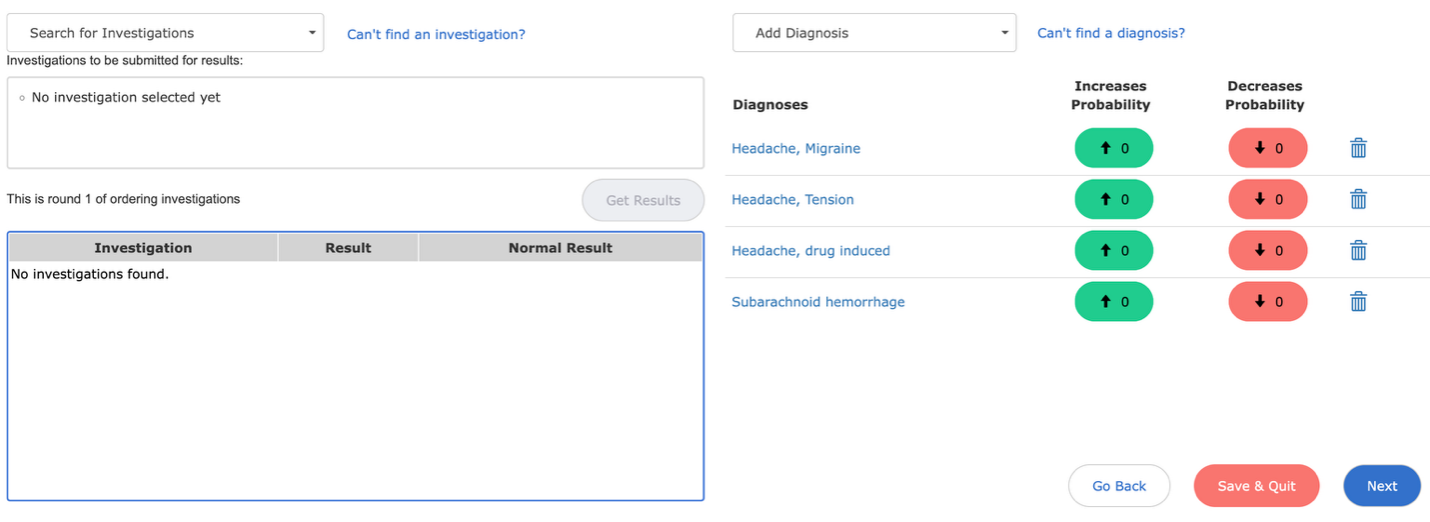
**

Students type and search for tests they want. Tests are queued for results:


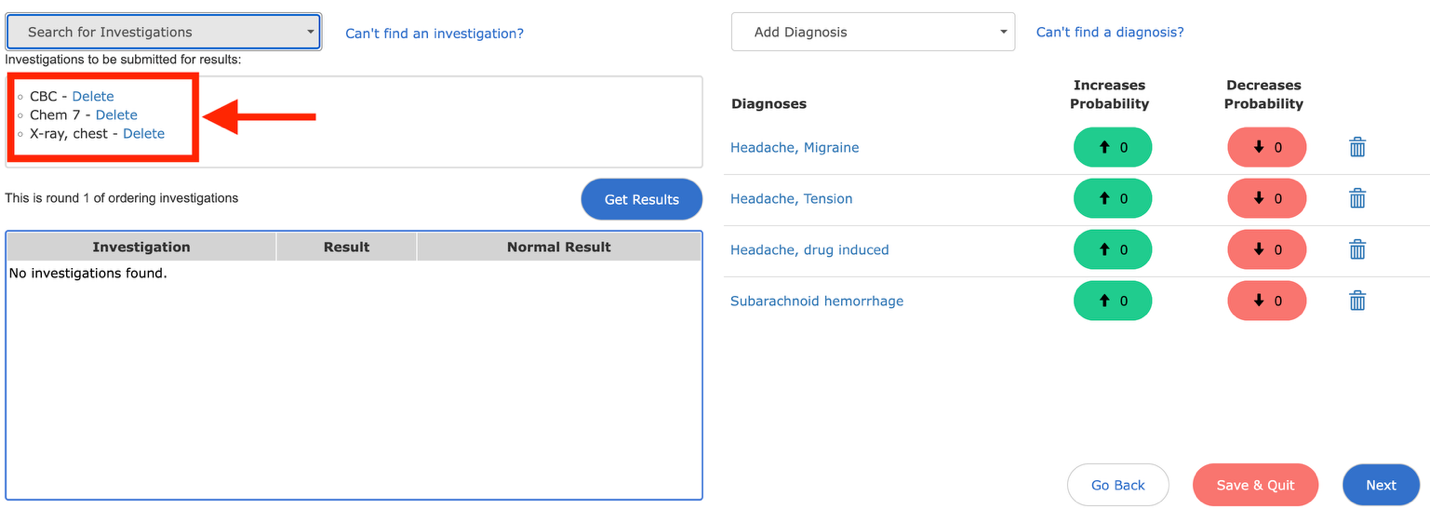


Click on “Get Results”. This defines 1 round of tests.
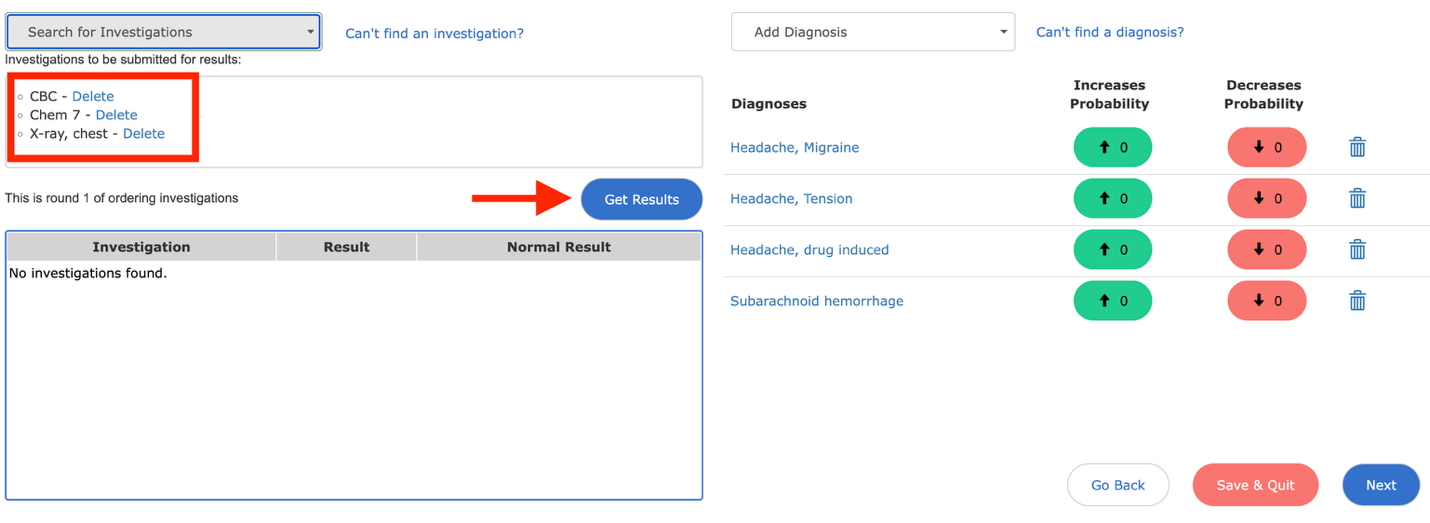


When test results are showing, they are highlighted so that the student can interact with them. Students assign the test results to the Ddx to increase or decrease probability, same as they did in the history and physical exam.

Here, the CXR report is expanded and data is clickable. The labs are clickable also to interact with the Ddx.


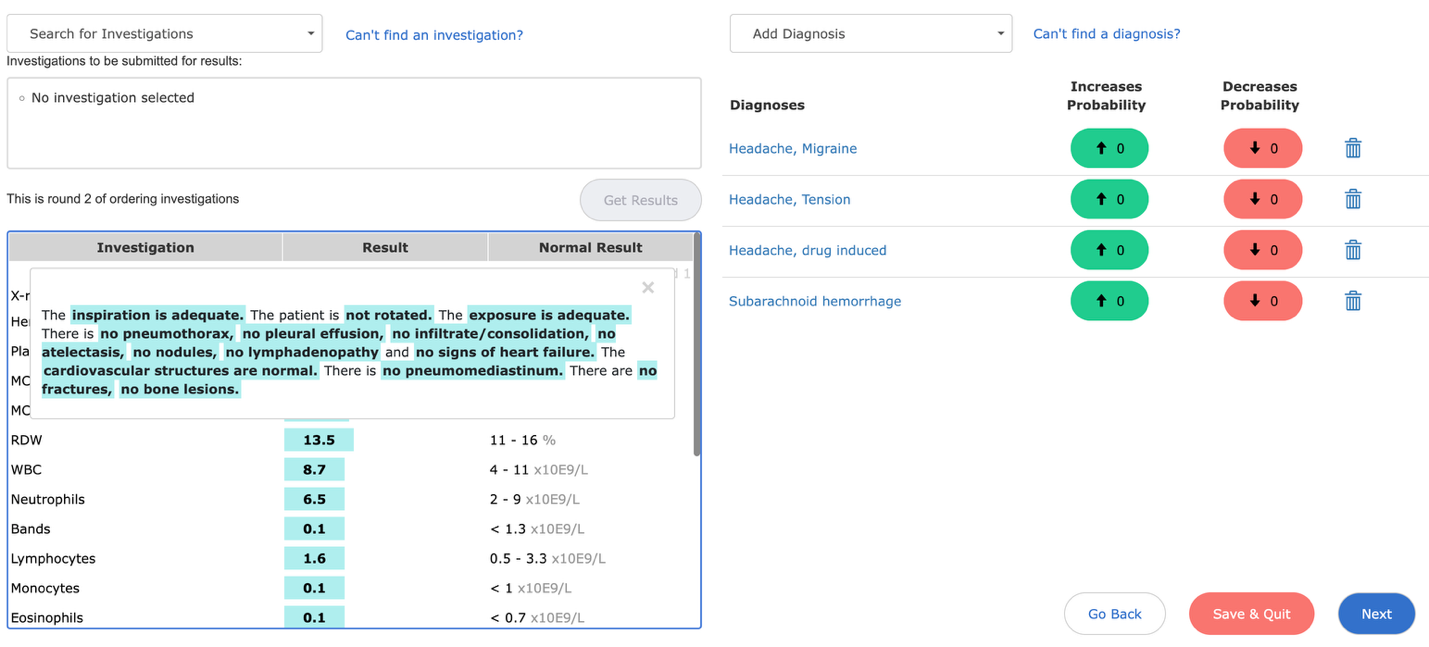


If students ordered more tests after this, these results would constitute round #2.

**Scoring and Feedback:** below are screenshots of feedback provided to students for one case.

**Ddx Feedback:** This student included 5 appropriate Dx’s to the Ddx and thus scored 100% (4 were required for full marks). Additional appropriate Dx’s are listed below.

**
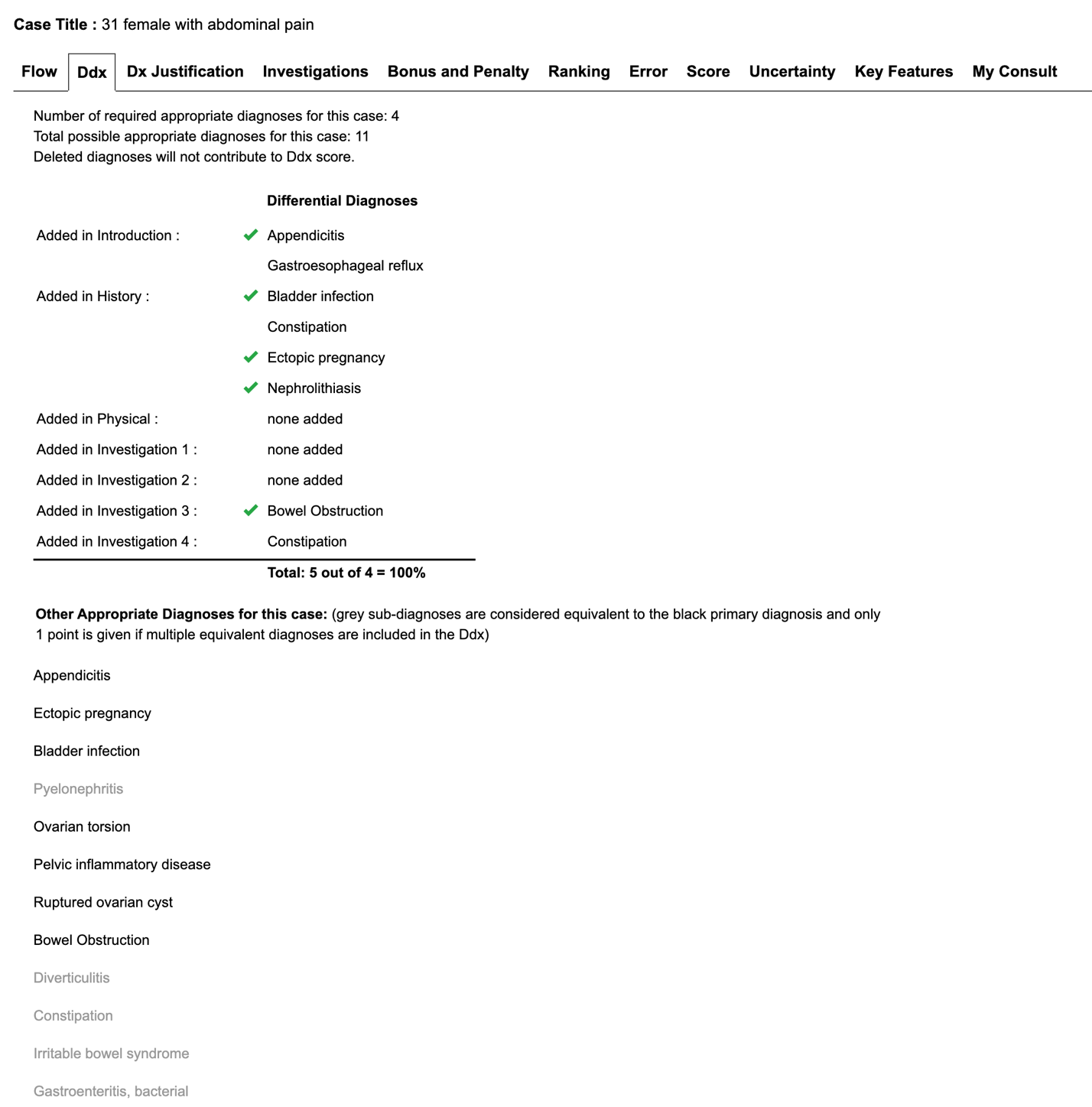
**

**Diagnostic Justification feedback:** there is a lot of data on this page. All data that the student clicked as increases or decreases for each diagnosis for this case is displayed and scored. Data is scored as:

- Earned a point (green checkmark)
- *Missed point* (item is in scorecard but not added by student)
- **Wrong** (item is actually wrong and student loses 0.5 points)
- Neutral (item is neither right nor wrong and scores no points)


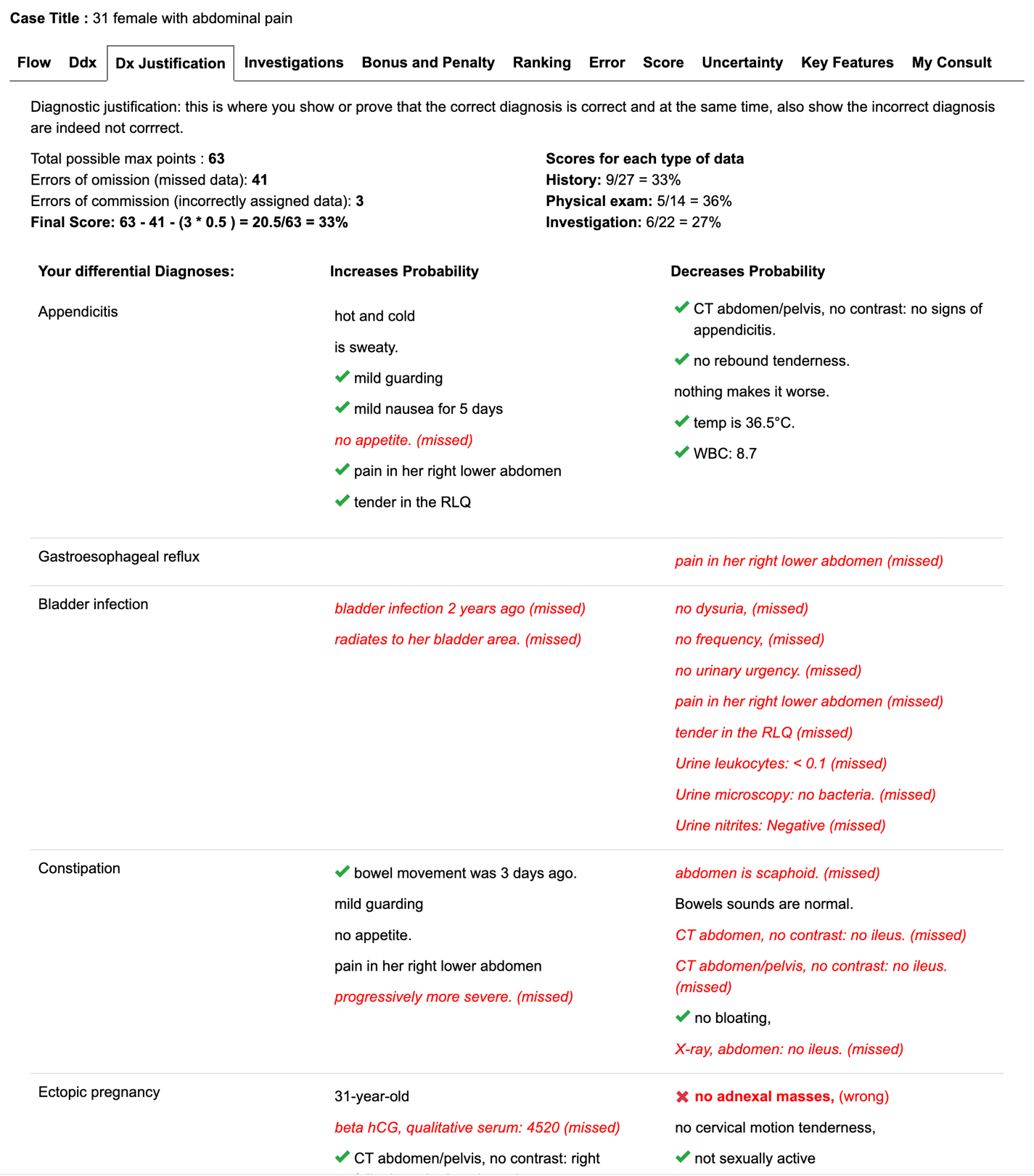


**Diagnostic Justification further details in above example:**

- Ectopic pregnancy was the correct dx.
- Quantitative scores are calculated at the top:
  - A breakdown of how well the history, physical exam and investigation data is analyzed is reported in the top right.
- In this example, the student correctly identified many clinical data that DECREASED the probability of appendicitis:
  - No fever
  - No elevated WBC
  - No rebound tenderness
  - Specifically, that there was no sign of appendicitis on the CT (same results would have been reported on an ultrasound as well, if ordered)
  - When we look at the most probable diagnosis (Appendix page 13), we see how the student appropriately did NOT rank appendicitis as most probable at the end of the case.
- We can also see ectopic pregnancy was in the Ddx, but there was no attempt to rule it out or confirm the diagnosis:
  - There was a positive pregnancy test; the student did not order this test (see Investigations screenshot below)
  - The student missed assigning this to the Dx ectopic pregnancy
  - the student also did not order this test (see Appendix page 12)
  - This is an example of Failure to Rule out (although if the student tried to rule out this diagnosis, they would have likely ruled it in)
- The student did not assign any data for bladder infection:
  - there was a lot of pertinent negative data that could have been assigned to DECREASES probability.
  - It appears that although this Dx was added to the Ddx, it was neglected in the justification process.

**Investigation Feedback:** this page shows which tests were ordered.
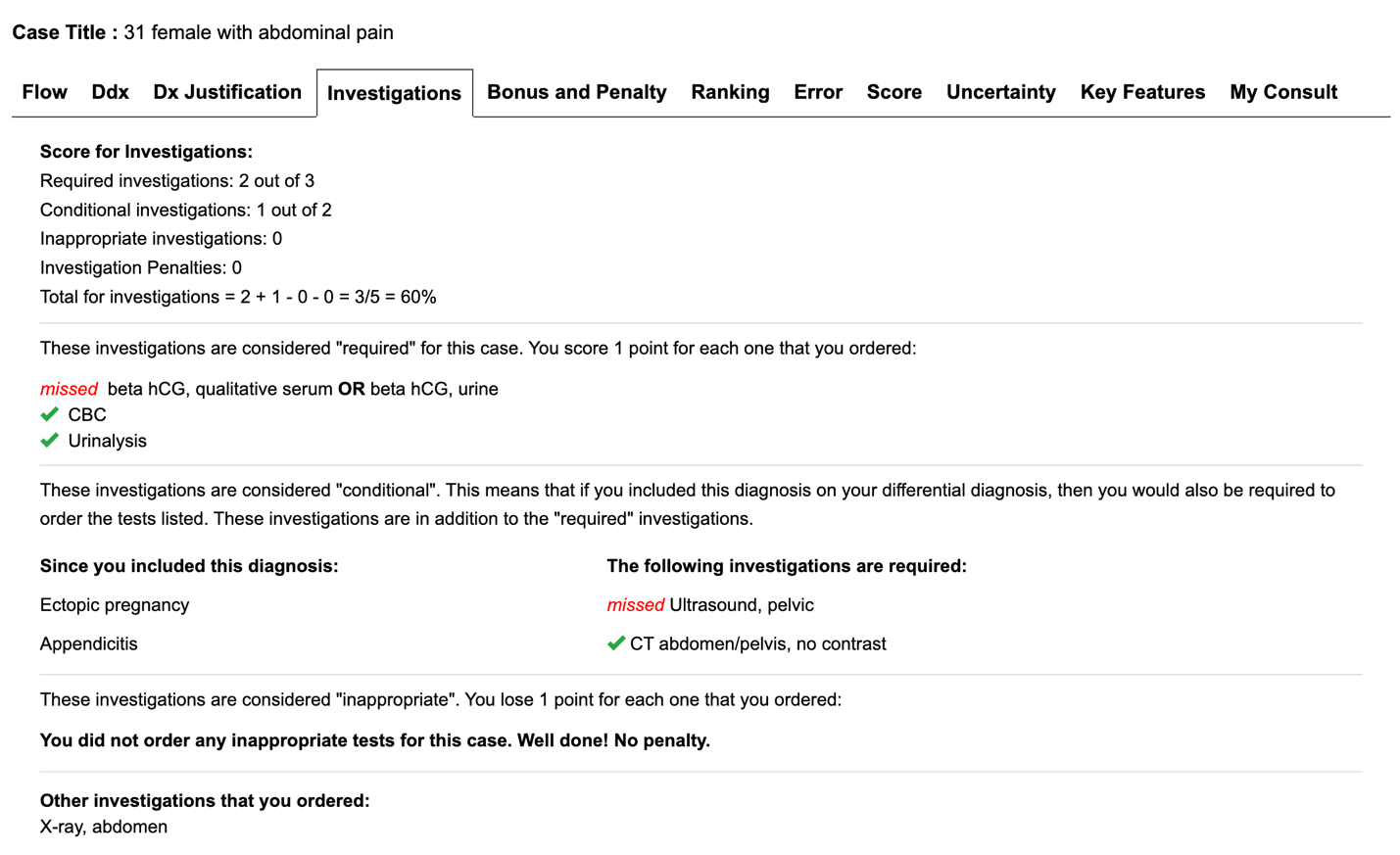


**Important observations here:**

- Required tests for this case included a bHCG (blood or urine); both were missed
- Ectopic pregnancy was on the Ddx and because of this, a pelvic ultrasound was also required for this patient (since she had a positive bHCG);
  - Had a pelvic U/S been ordered, it would have revealed no products on conception in the uterus and a swollen right fallopian tube
- we can now begin to understand the cause of the misdiagnosis; there was a failure of diagnostic justification in association with a failure of data collection in this case.

**Most Probable Diagnoses:** this shows the most probable diagnoses assigned at each stage.


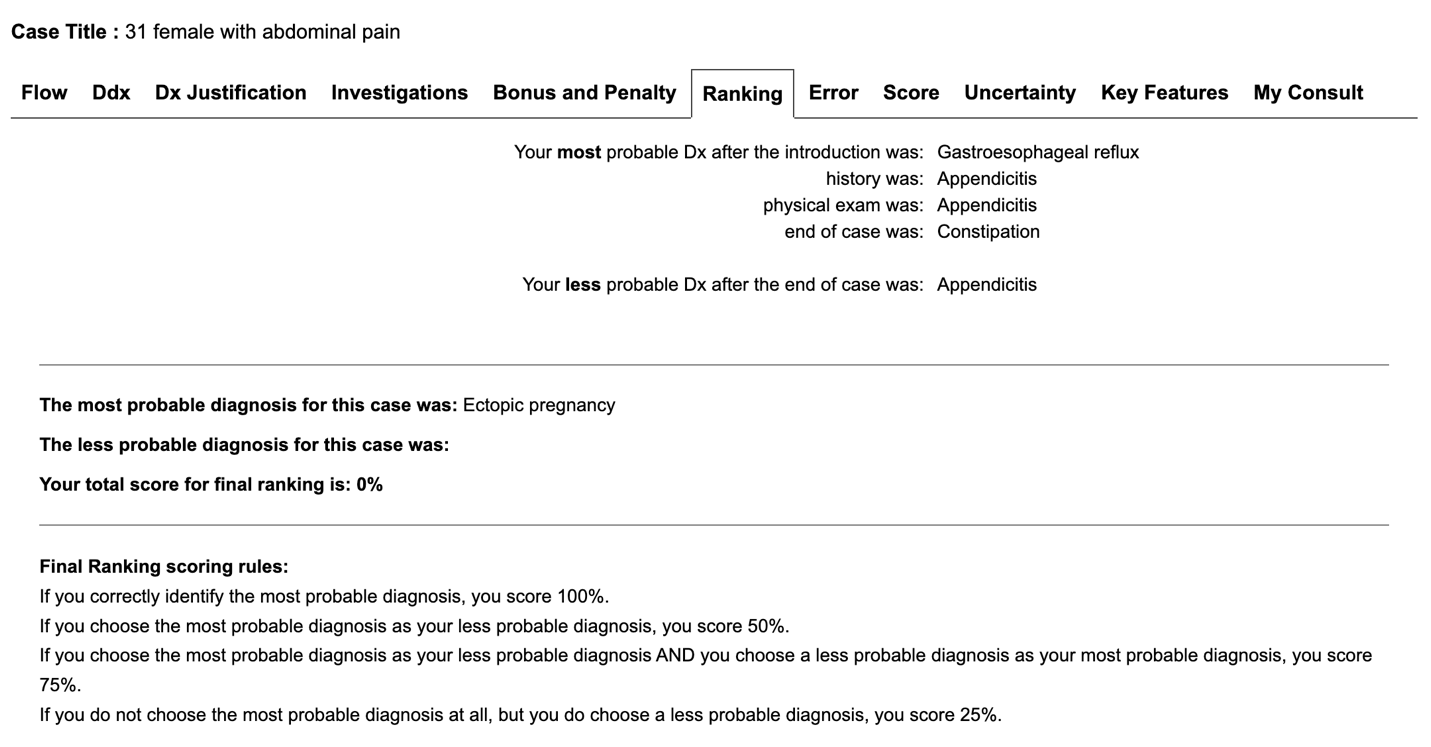


- The student appropriately prioritized the diagnosis of appendicitis after the history and physical exam, prior to the test results.
- However, when appendicitis was ruled out, the student did not revisit all Dx’s in the Ddx and missed the important one to rule out: ectopic pregnancy.

**Total score for case:**

**
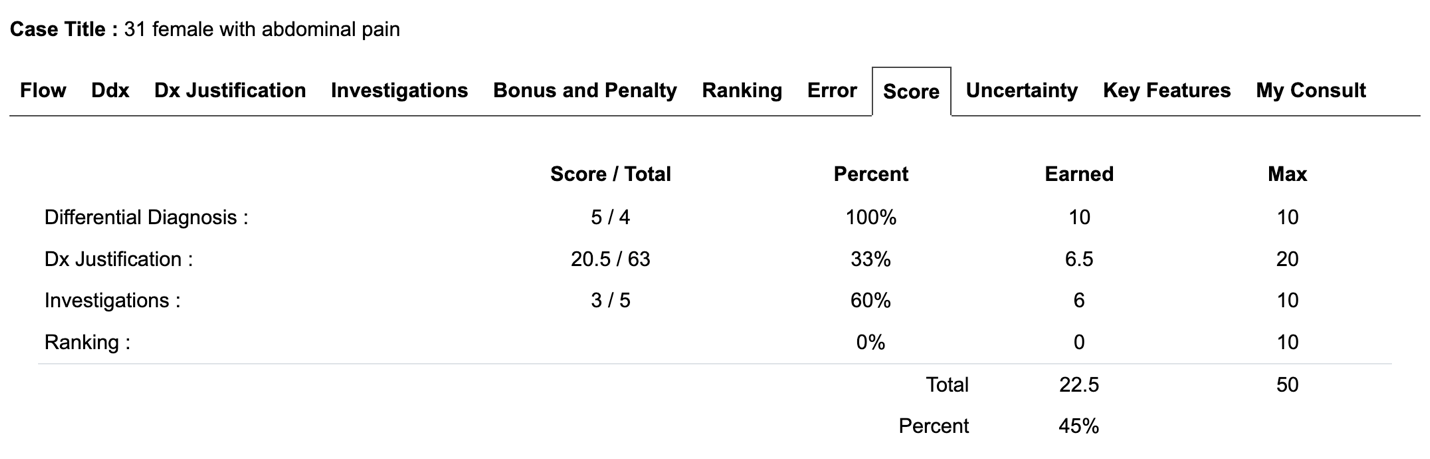
**

A quantitative score for each of the 3 components of diagnostic reasoning are provided and a score for diagnostic accuracy. Diagnostic justification is weighted double (out of 20 instead of 10) to highlight its importance in the role of diagnostic accuracy.

**Instructor View of Misdiagnoses:** the instructor has an over-arching view of misdiagnosis stats


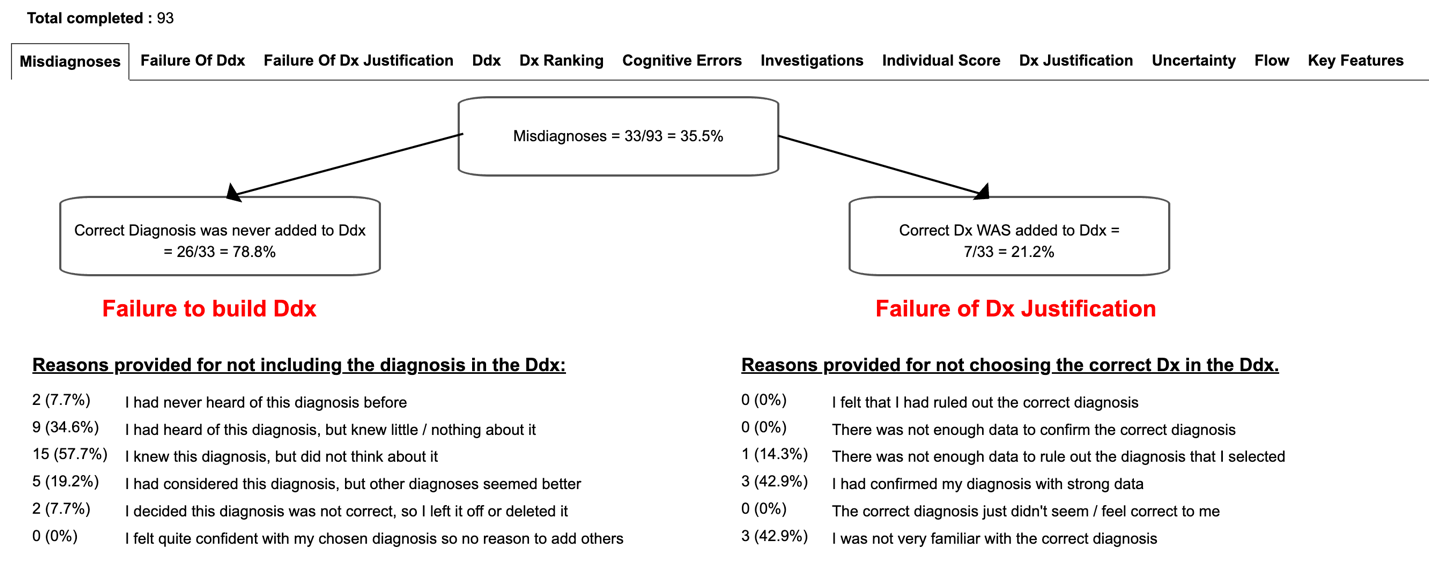


Insights into how many misdiagnoses occurred in the class:

- How many students never included the correct Dx in the Ddx (failure to build Ddx)
- How many students included the correct Dx, but did not choose it (failure of DxJ)
- We see that nearly 78.8% of the misdiagnosed cases never had the correct Dx added to the Ddx.
- Students self-reported reasons why THEY thought they misdiagnosed the case:
  - Answers fall under the categories of knowledge deficit vs. reasoning error
  - Among failure to build Ddx, we see 7.7% + 34.6% = 42.3% indicated lack of knowledge
  - while 57% + 19.2% + 7.7% = 83.9% indicated reasoning errors.
  - Students can select multiple choices.

**Instructor View all students:** Instructors can view all students and see the same detailed information each student receives.


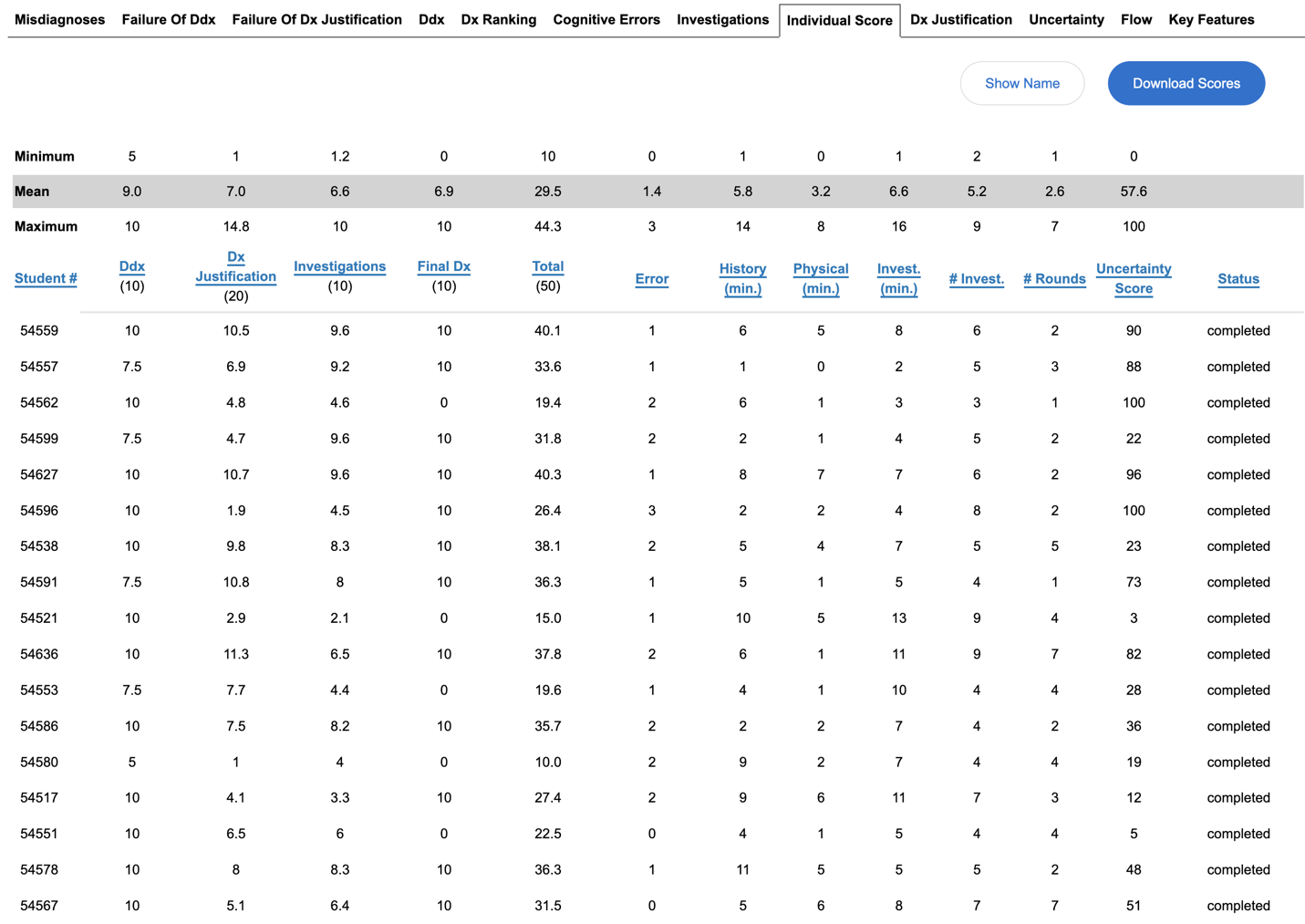


For additional information on stats and feedback, instructors can [view this video](https://drive.google.com/file/d/18A7t2XsLyaIYjZktQfQMTR2VWBCaRbhG/view).

**Cognitive errors**

**Premature closure (failure to confirm the correct diagnosis):** if the student identifies the correct diagnosis for the case AND they did not assign specific confirmatory clinical and/or test result data as **INCREASES PROBILITY**, then they were identified as having behavior consistent with premature closure.

- Example: to confirm a pulmonary embolism, students were required to: 1) include pulmonary embolism in the Ddx **AND** 2) perform a CT angiogram (CTA/CTPE) **AND** 3) assign “there is a clot in the right PA” as **INCREASES PROBILITY** to pulmonary embolism. A positive D-dimer was not accepted as confirmation of the diagnosis; nor was a CT without contrast (since the report for this study did not mention any clot).

**Failure to rule out:** dangerous or “must not miss” diagnoses were identified for each case. If the student did not assign specific clinical and/or test result data as **DECREASES PROBABILITY**, then they were identified as having behavior consistent with failure to rule out. If this diagnosis is not included in the Ddx, then they were also identified as having behavior consistent with failure to rule out.

- Example: to rule out an ectopic pregnancy, students were required to: 1) have ectopic pregnancy in the Ddx, **AND** 2) perform a pregnancy test **AND** 3) assign the negative result pregnancy result as **DECREASES PROBABILITY** for ectopic pregnancy

**Anchoring:** at the end of the introduction (triage stage), history, physical exam, and the end of the case (after investigations), the student is asked what their most probable diagnosis is. If the student identifies the same diagnosis at 2 or 3 of the first 3 stages of the case AND at the end of the case, then they are identified as having behavior consistent with anchoring.

- Example: in a case where the diagnosis is **NOT** bacterial pneumonia, **AND** the student indicates bacterial pneumonia as their most probable diagnosis at the end of the case **AND** indicated bacterial pneumonia as their most probable diagnosis at 2 of 3 of the stages during the case.
- Note: if the student identified the CORRECT diagnosis as their most probable diagnosis, they are not flagged for anchoring.

**Confirmation Bias:** if a student is identified as anchoring AND the student did NOT assign obvious clinical and/or test result data that rules the diagnosis out as **DECREASES PROBABILITY**, then they are identified as having behavior consistent with confirmation bias.

- Example: the correct diagnosis is bacterial pneumonia and the student is flagged for anchoring for pulmonary embolism **AND** the student did **NOT** assign “no pulmonary embolism seen” from the CT angiogram as **DECREASES PROBABILITY** to pulmonary embolism.

**Search Satisficing:** if a student identifies an intermediary (but correct) diagnosis as the final most probable diagnosis, but this diagnosis is NOT the root cause of the problem, then they are identified as having behavior consistent with search satisficing.

- Example: A male patient presents with severe iron deficiency anemia and chronic melena from colon cancer and the student completes the case with a diagnosis of anemia.

***Note:*** Assigning clinical data as increasing or decreasing the probability of a diagnosis is diagnostic justification. This process is central to 3 of the 5 cognitive error algorithms and highlights the association between diagnostic justification and cognitive error rates.

Graphs of each cohort’s psychometric performance:


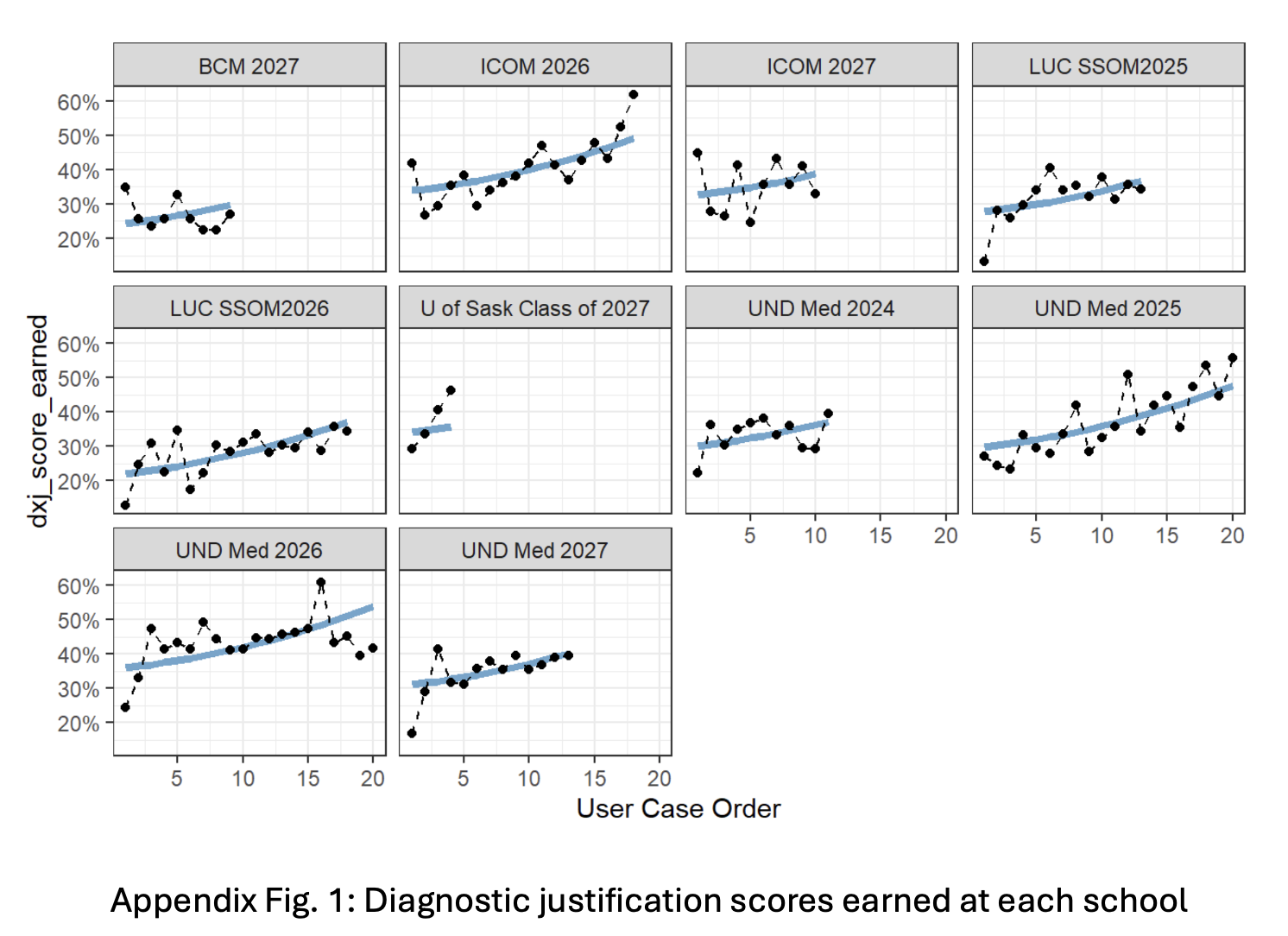


**Appendix Fig 1**: for each cohort, scores for diagnostic justification is plotted versus the number of cases completed.


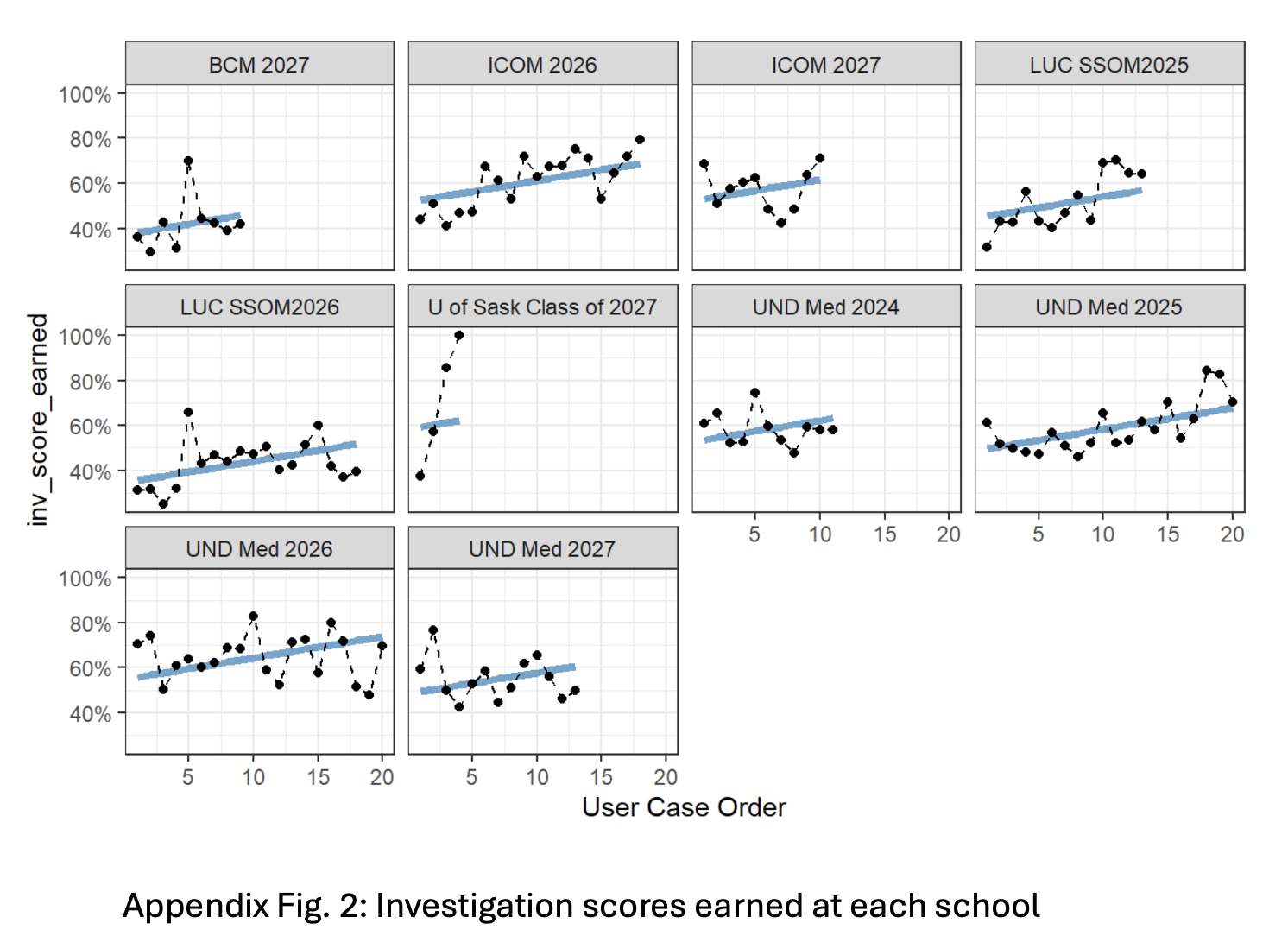


**Appendix Fig 2**: for each cohort, scores for ordering tests is plotted versus the number of cases completed.


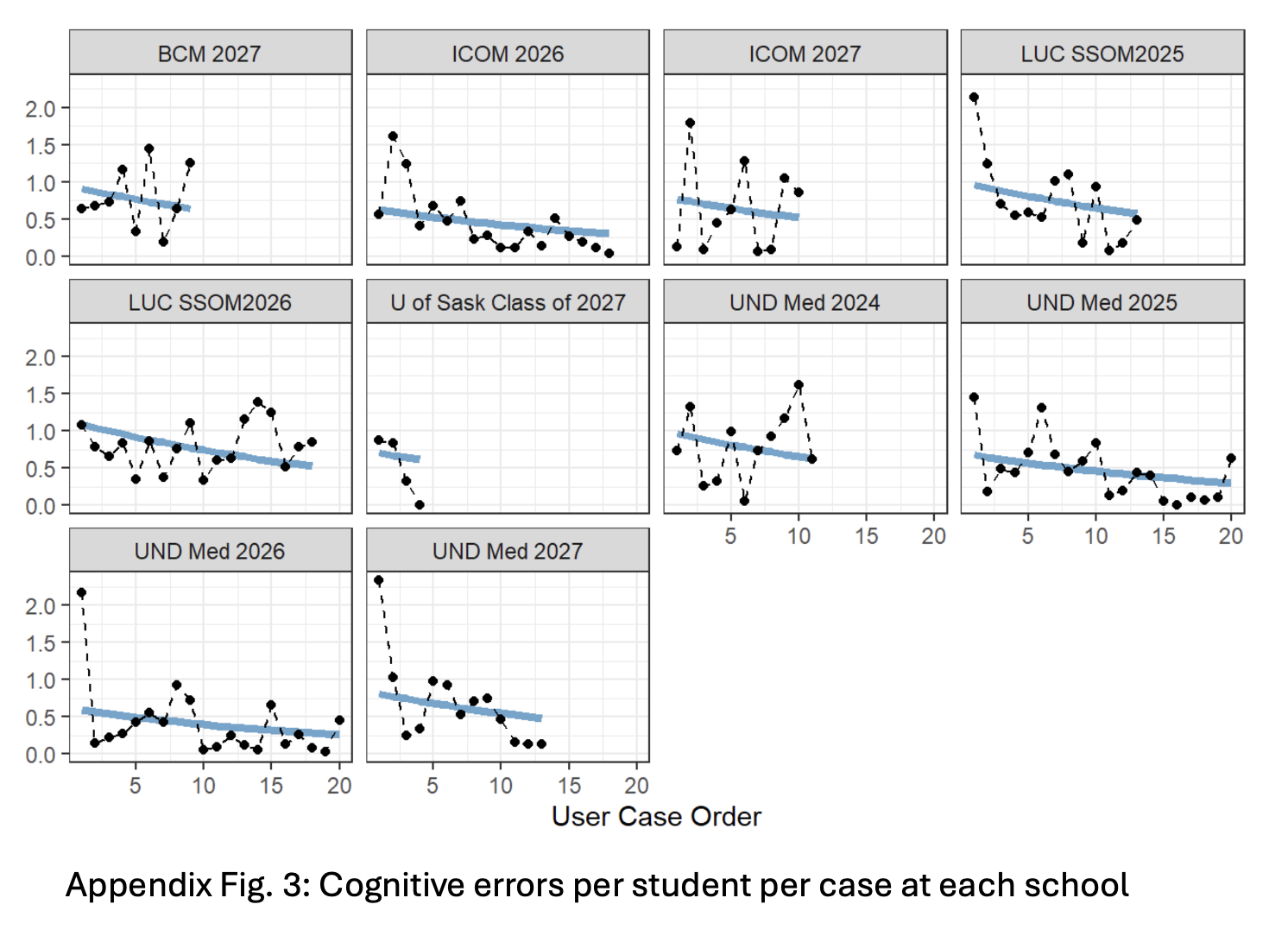


**Appendix Fig 3**: for each cohort, the count of cognitive errors per student per case is plotted versus the number of cases completed.


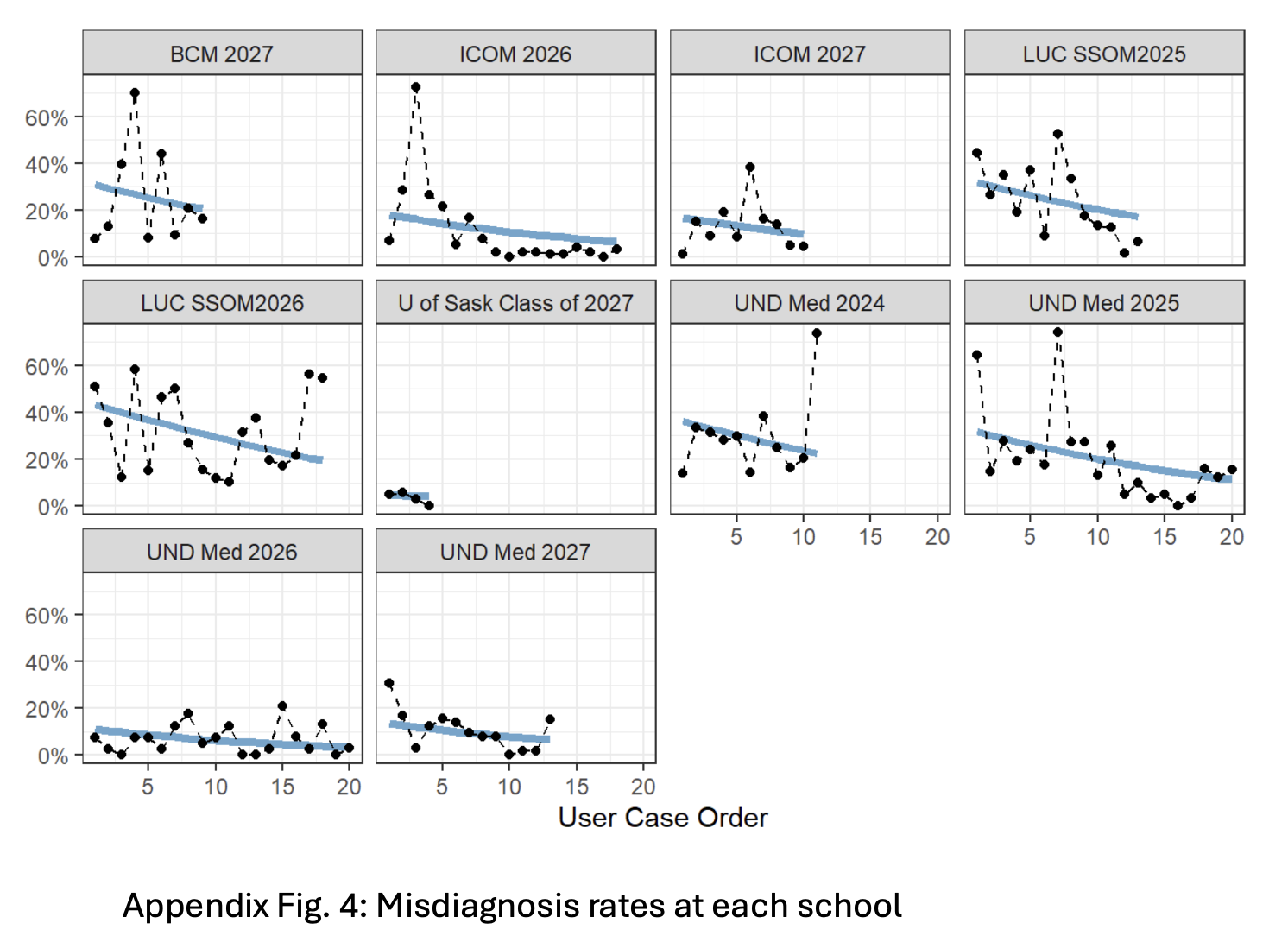


**Appendix Fig 4**: for each cohort, misdiagnosis rates are plotted versus the number of cases completed.
